# Developing a Library of Mannose-Based Mono- and Disaccharides: A General Chemoenzymatic Approach to Monohydroxylated Building Blocks

**DOI:** 10.3390/molecules25235764

**Published:** 2020-12-07

**Authors:** Lisa Tanzi, Marina Simona Robescu, Sara Marzatico, Teresa Recca, Yongmin Zhang, Marco Terreni, Teodora Bavaro

**Affiliations:** 1 Department of Drug Sciences, University of Pavia, viale Taramelli 12, I-27100 Pavia, Italy; lisa.tanzi01@universitadipavia.it (L.T.); marinasimona.robescu@unipv.it (M.S.R.); sara.marzatico01@universitadipavia.it (S.M.); marco.terreni@unipv.it (M.T.); 2 Institut Parisien de Chimie Moléculaire, Sorbonne Université, CNRS, UMR 8232, 4 Place Jussieu, 75005 Paris, France; yongmin.zhang@upmc.fr; 3 Centro Grandi Strumenti, University of Pavia, via Bassi 21, I-27100 Pavia, Italy; teresa.recca@unipv.it

**Keywords:** acetyl xylan esterase, *Candida rugosa* lipase, enzyme immobilization, enzymatic hydrolysis, monosaccharides, disaccharides, mannose-based oligosaccharides

## Abstract

Regioselective deprotection of acetylated mannose-based mono- and disaccharides differently functionalized in anomeric position was achieved by enzymatic hydrolysis. *Candida rugosa* lipase (CRL) and *Bacillus pumilus* acetyl xylan esterase (AXE) were immobilized on octyl-Sepharose and glyoxyl-agarose, respectively. The regioselectivity of the biocatalysts was affected by the sugar structure and functionalization in anomeric position. Generally, CRL was able to catalyze regioselective deprotection of acetylated monosaccharides in C6 position. When acetylated disaccharides were used as substrates, AXE exhibited a marked preference for the C2, or C6 position when C2 was involved in the glycosidic bond. By selecting the best enzyme for each substrate in terms of activity and regioselectivity, we prepared a small library of differently monohydroxylated building blocks that could be used as intermediates for the synthesis of mannosylated glycoconjugate vaccines targeting mannose receptors of antigen presenting cells.

## 1. Introduction

Oligosaccharides are involved in the modulation of biomolecules properties and in the communication between cells; thus, they play important roles in a variety of physiological and pathological processes, such as cell growth and proliferation, angiogenesis, protein folding and degradation, cell-cell communication, cell-pathogen interactions and immune response [1]. In human cells, oligosaccharides often occur as glycoconjugates attached to other macromolecules, such as lipids (glycolipids) and proteins (glycoproteins) [2]. Glycoconjugates can promote the immune response through the production of antibodies against a specific target; therefore, their use as vaccines has been proven to be a successful strategy to prevent infectious diseases [3].

Mannose-based glycoconjugates were investigated for improving antigens up-take mediated via the mannose receptor (MR) of human antigen presenting cells (APCs). MR belongs to C-type lectin receptor family and it has numerous functions, including the recognition of glycans present on microorganisms surface and the consequent antigen internalization by phagocytosis and presentation [4]. The discovery of MR role in antigen up-take stimulated the research towards the development of mannosylated antigens with the aim to design vaccines with improved immunogenicity by targeting MR [5].

Several studies regarding natural high-mannosylated glycoprotein120 (GP120) fragments as potential human deficiency virus (HIV) vaccine were previously reported [6,7]. In HIV, high-mannose glycans with α-(1→6) and α-(1→2) motifs are included in the important envelope glycoprotein GP120, involved in the mediation of the infection process by interacting with MR exposed on APC cells surface. Unfortunately, this approach resulted poorly efficient for the development of an effective HIV vaccine candidate, but it suggested the idea that mannosylation with natural polymannan or its analogues, being well-recognized by MR of APC, represents a possible strategy to improve the antigenic activity of peptides or proteins. Similarly, different synthetic α-(1→6) polymannan, mimicking *Mycobacterium tuberculosis* polysaccharides, were synthesized and characterized showing good binding affinity for lectins [8].

However, the structural diversity and complexity of natural oligosaccharides, including polymannan, make their production from natural sources and characterization extremely complicated. Thus, chemical and/or enzymatic synthesis can represent a valid alternative for the production of structure-defined oligosaccharides analogues in high purity, by exploiting efficient and scalable protocols [9]. Automatized methods either chemical or enzymatic have been developed over the years for the synthesis of oligosaccharides [10,11], including an α-(1→6)-30mer-mannoside [12]. However, the preparation of sugar acceptor building blocks bearing only one free hydroxyl group in the desired position represents the main bottleneck in the synthesis of oligosaccharides. Usually, the chemical synthesis of monodeprotected monosaccharides and disaccharides is performed through orthogonal multistep processes, which frequently result in low yields [13,14].

The use of enzyme-catalyzed reactions provides, instead, a more straightforward route: hydrolases have been successfully employed as catalysts in the regioselective deprotection of peracetylated mono- [15,16,17,18] and disaccharides [19,20,21] under mild reaction conditions. Hence, lipases (EC 3.1.1.3) and esterases (EC 3.1.1.x) represent valuable tools for a simple and efficient chemoenzymatic approach in the preparation of sugar building blocks involving the use of acetyl moiety as the only protecting group [21,22]. In particular, the availability of synthetic tools for the production of acetylated mannose building blocks with only one free hydroxyl group and different reactive groups in anomeric position would facilitate the preparation of polymannan analogues with α-(1→6) and α-(1→2) motifs and the subsequent biological evaluation of their corresponding glycoconjugates derivatives.

In this context, the use of immobilized hydrolases is highly sought since peracetylated sugars, especially disaccharides, are scarcely soluble in aqueous medium; thus, the use of organic co-solvents is required. Stabilization of enzymes via immobilization techniques is a valuable strategy to enhance the stability of biocatalysts in the presence of organic co-solvents and their easy recovery from reaction mixture and re-use [23]. The immobilization of lipases on hydrophobic supports, by means of interfacial adsorption (e.g., on octyl-Sepharose or octadecyl-Sepabeads), is a well-established methodology for obtaining highly active biocatalysts [24] with good stability in the presence of organic co-solvents [25].

Conversely, this simple methodology is not suited for esterases due to their different 3D-architecture and kinetics [26,27]. However, the esterase fraction from the crude extract of *Aspergillus niger* lipase (ANL) and acetyl xylan esterase from *Bacillus pumilus* (AXE) were successfully immobilized by covalent interaction on acrylic carriers and employed for the deprotection of acetylated mono- and disaccharides [15,19,21].

In the present work we report on a comparison between immobilized *Candida rugosa* lipase (CRL) and acetyl xylan esterase from *Bacillus pumilus* (AXE) in the synthesis of monohydroxylated sugar building blocks. A new immobilization protocol based on agarose carrier was developed for AXE to obtain a robust biocatalyst under a wide range of experimental conditions. An extensive screening of acetylated mannose-based mono- and disaccharides differently functionalized in C1 position provided a library of monodeprotected intermediates useful for the synthesis of mannosylated glycoproducts.

## 2. Results and Discussion

### 2.1. Immobilization of Acetyl Xylan Esterase from Bacillus Pumilus and Stability Studies

**Figure 1:**
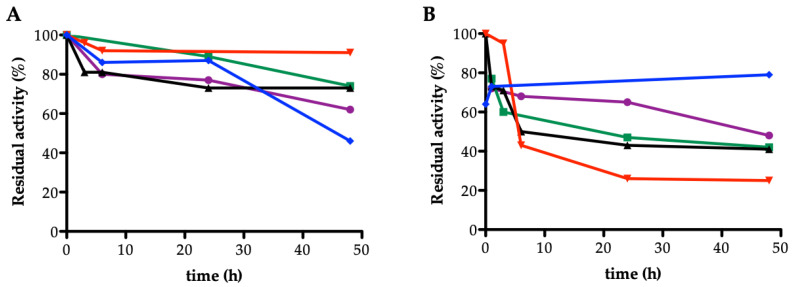
Stability of soluble acetyl xylan esterase from *Bacillus pumilus* (AXE) (**A**) and immobilized AXE on acrylic epoxy resin (**B**) in different organic co-solvents at different percentages *v*/*v*: 15% acetonitrile (purple circles), 30% acetonitrile (green squares), 50% acetonitrile (black triangles), 90% ethanol (red triangles), 90% tert-butanol (blue rhombus). The measurements were performed in duplicate.

Considering that the acetylated monosaccharides and disaccharides are not completely soluble in buffer, we evaluated the stability of soluble acetyl xylan esterase from *Bacillus pumilus* (AXE) in different types of co-solvents present in the reaction mixture at different percentages *v*/*v* (Figure 1A). Soluble AXE was quite stable in all the reaction mixtures tested. After 48 h of incubation in 90% ethanol, the enzyme maintained a residual activity of 90%. However, in the presence of acetonitrile and *tert*-butanol, the biocatalyst showed lower stability after 48 h incubation (Figure 1A). Thus, in order to obtain a robust biocatalyst, different immobilization carriers were evaluated.

Immobilized AXE on acrylic epoxy resin was already successfully employed for the selective deprotection of peracetylated lactose and *N*-acetyl glucosamine derivatives [15,21]; however, a low stability profile of the biocatalyst during the reaction course was observed. As reported in Figure 1B, the residual activity of immobilized AXE on acrylic epoxy resin after 48 h incubation is lower than 60% in all conditions tested, except 90% *tert*-butanol, in which the enzyme maintained between 70%–80% residual activity.

Thus, a new immobilization protocol was set-up for AXE considering its multimeric three-dimensional structure. In fact, AXE has a complex hexameric quaternary structure formed by a dimer of trimers showing a “doughnut-shaped” assembly with the six active centers disposed towards a central pore [28,29]. When working with multimeric enzymes, the immobilization technique must be able to bind all the subunits in order to keep the quaternary structure of the enzyme unmodified (or poorly modified) under the selected experimental conditions. The potential dissociation into the single constitutive monomers can cause the loss of activity. This dissociative process is usually enhanced by extreme pH values, high temperature, and the presence of organic co-solvents, conditions often necessary to ensure the solubility of poorly water soluble substrates [30].

We decided to use glyoxyl-agarose (GLX-AG), a hydrophilic carrier activated with aldehyde functional groups, for AXE immobilization. GLX-AG has a wide hydrophilic superficial area and pores of such dimensions to harbor proteins in a wide range of molecular weights. In addition, the presence of several hydroxyl groups on its surface, that can be easily activated, allows for the formation of a high number of bonds between the enzyme and the carrier resulting in a three-dimensional network of covalent multipoint interactions with consequent stabilization effects [31]. Successful immobilization procedures for multimeric proteins stabilization based on this carrier have been widely described [32,33].

Different parameters were screened, such as temperature, time, and protein loading in order to optimize AXE immobilization on GLX-AG. As shown in Table 1, when using a loading of 150 mg/g and 25 °C, AXE immobilized on acrylic epoxy resin showed higher immobilization yields in terms of quantity of immobilized protein (39%) and activity (38%) when compared to GLX-AG derivative (28% of protein and 33% of activity immobilized); however, the activity of this derivative was almost four-fold lower (107 U/g vs. 383 U/g). Thus, it seems that the hydrophobic microenvironment of acrylic surface around the enzyme negatively influences its activity, while the hydrophilic nature of agarose seems to be preferred. Moreover, the multipoint interaction of the enzyme with the three-dimensional network of agarose fibers may prevent subunits dissociation maintaining the correct quaternary structure of the protein and, thus, its activity. In addition, the short time incubation needed for GLX-AG immobilization protocol compared to acrylic resin immobilization (3 h instead of 24 h) can explain the better performances of GLX-AG biocatalyst. We further tried to optimize GLX-AG immobilization procedure by lowering the temperature to 4 °C in order to prevent subunits dissociation during the immobilization process. However, as shown in Table 1, we were able to slightly increase the immobilization yield in terms of activity (48%), but the final activity expressed by the derivative was lower compared to the immobilization procedure performed at 25 °C (283 U/g vs. 383 U/g). The longer incubation time (18 h instead of 3 h) at pH 10 may negatively influence the stability of the enzyme. Finally, as in all the conditions tested we have observed low protein binding to the supports (26%–39% of the total protein used), we decided to decrease the immobilization loading from 150 mg/g to 50 mg/g. In these conditions, we obtained a two-fold increase in the % of immobilized protein (61% vs. 28%) and an improvement of immobilized activity (43% vs. 33%). Thus, the best immobilization conditions on GLX-AG were 25 °C, 3 h using 50 mg/g of protein. This biocatalyst was further used in all the experiments reported in this paper.

**Figure 2:**
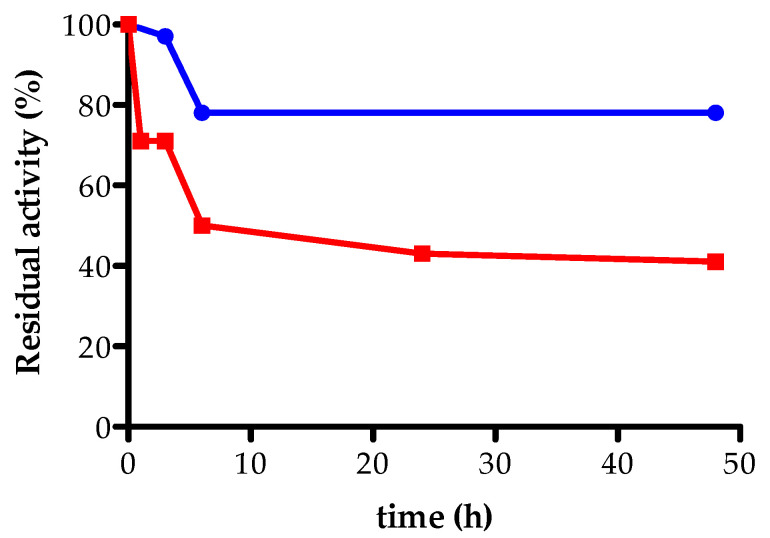
Stability of immobilized AXE on acrylic epoxy resin (red squares) and on glyoxyl-agarose (GLX-AG) (blue circles) in 50% acetonitrile. The measurements have been performed in duplicate.

The stability of AXE immobilized on GLX-AG was evaluated in 50% acetonitrile and phosphate buffer (100 mM; pH 7.0), and it was compared to that of the derivative immobilized on acrylic resin. As shown in Figure 2, AXE immobilized on GLX-AG showed higher stability compared to the acrylic resin derivative: GLX-AG derivative maintained 80% of its activity after 48 h of incubation, while the acrylic resin derivative lost almost 50% of its activity, in the same conditions.

### 2.2. Enzymatic Hydrolysis of Peracetylated Monosaccharides (***1**–**8***)

**Scheme 1:**
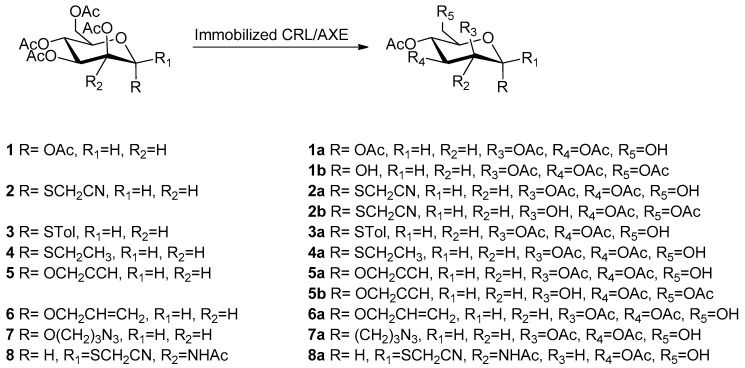
Regioselective enzymatic hydrolysis of peracetylated mannopyranosydes **1**–**7** and *N-*acetyl glucosamine (**8**) derivatives.

The enzymatic regioselective hydrolysis of different peracetylated d-mannopyranoses (**1**–**7**) and one glucosamine derivative (**8**) (Scheme 1) was studied by using *Candida rugosa* lipase (CRL) immobilized on octyl-Sepharose^®^ (OC-AG) and acetyl xylan esterase from *Bacillus pumilus* (AXE) immobilized on glyoxyl-agarose (GLX-AG) in order to screen the regioselectivity of the two biocatalysts. The results are shown in Table 2. CRL was highly selective hydrolyzing preferably the primary position (C6) for compounds **1**, **3**, **4**, **5**, **6**, and **7** forming, respectively, **1a** [17], **3a** [34], **4a**, **5a**, **6a**, and **7a** [34] with good yields (50%–81%). Compound **2** was deacetylated by CRL simultaneously in position C6 and C2, giving access to **2a** (50%) and **2b** (40%), both useful building blocks for further oligosaccharides synthesis [17]. Compared to CRL, AXE seemed less specific for the same compounds, showing the same regioselectivity as CRL but lower yields (24%–50%) due to the formation of undesired by-products.

Interestingly, for compound **5**, CRL and AXE showed a different regioselectivity profile: CRL selectively deprotected the primary position (C6) giving **5a** (80%), while AXE preferred the C2 position, giving access to **5b** in acceptable yields (40%). These results highlighted a different and complementary selectivity behavior of the two biocatalysts towards the same substrate.

Compound **8** was selectively hydrolyzed by using AXE immobilized on GLX-AG, and the results were in agreement with those previously reported using the enzyme immobilized on acrylic epoxy carrier [15,35].

These monodeprotected products are useful building blocks in the synthesis of oligosaccharides and glycoconjugates. For example, compounds obtained from **2a** and **8a** can be used to conjugate antigenic proteins through the thiocyanomethyl group in anomeric position. Compound **3a** can be used both as acceptor or donor in further glycosylation reactions because, once used as acceptor, the product obtained can become a donor due to the good properties of S-Tol group as leaving group. Furthermore, compounds obtained by hydrolysis of **5** and **7** can be used as intermediates to prepare glycoproducts by click chemistry, thanks to their propargyl and azido groups in the anomeric position.

### 2.3. Synthesis of Peracetylated Disaccharides (***10**–**18***)

**Scheme 2:**
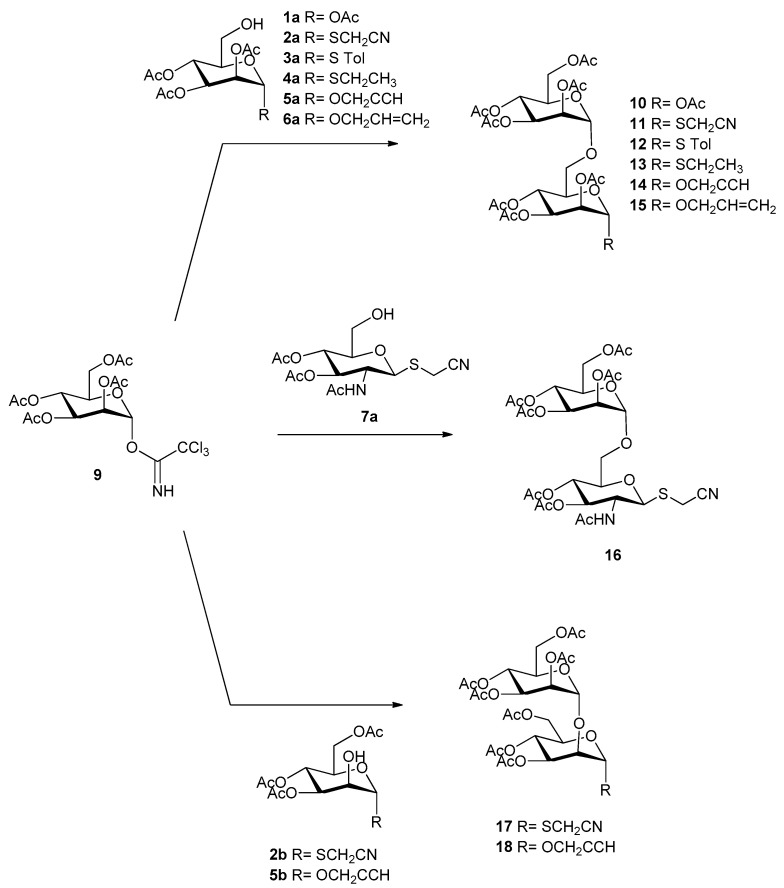
Synthesis of peracetylated mannose-based disaccharides (**10**–**18**). Reaction conditions are reported in Table 3.

Monodeprotected monosaccharides obtained previously were further used as sugar acceptors to synthesize different disaccharides by Schmidt glycosylation reaction (Lewis acid-catalyzed glycosylation using glycosyl trichloroacetimidate as donor as reported in Scheme 2). The reaction was optimized with respect to temperature and time depending on the glycosylation position (Table 3). Man(1→6)man disaccharides (**10**, **12**–**15**) were obtained in a range of 50% and 92% yield (except for **13**: 37% yield) by using 0 °C after 2.5–4 h. The same conditions were used also for compound **16** obtained in very good yield (74%). The synthesis of man(1→6)manSCH_2_CN (**11**) and man(1→2)man disaccharides required a very cold environment (−63/−70 °C) to afford products **11**, **17**, and **18** in 79%, 52%, and 80% yield, respectively (Table 3).

Thus, through the above described chemoenzymatic approach, a library of nine mannose-based disaccharides (**10**–**18**) with 1→6 or 1→2 linkage (Scheme 2), and with different anomeric reactive groups, were obtained with good yields and using only the acetyl group for protection.

### 2.4. Enzymatic Hydrolysis of Peracetylated Disaccharides (***10**–**18***)

The disaccharides prepared as previously reported (see Section 2.3) were subsequently tested as substrates for enzymatic hydrolysis with the aim to obtain selectively deprotected disaccharides that can be further used as advanced building blocks for the synthesis of complex linear and branched oligosaccharides. Both CRL and AXE were tested as catalysts (Table 4), but only AXE showed a relevant activity towards the different disaccharides **10**–**18** (CRL was almost completely inactive towards all these substrates).

**Scheme 3:**
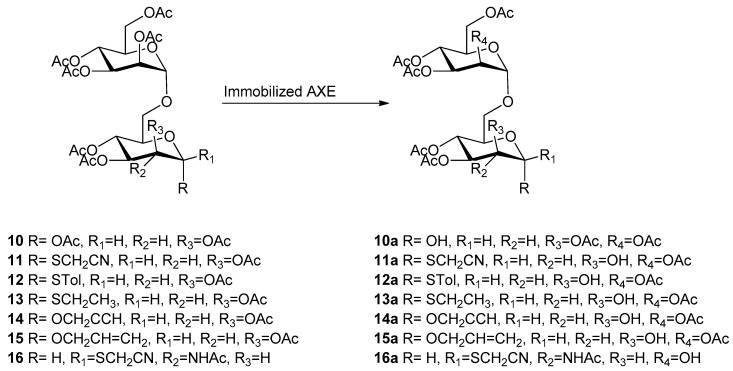
Regioselective enzymatic hydrolysis of peracetylated 1→6 disaccharides (**10**–**16**).

The hydrolysis catalyzed by AXE-GLX-AG derivative provided different results depending from the substrate as shown in Table 4. The fully acetylated man(1→6)man **10** (Scheme 3) was deprotected at the anomeric position yielding product **10a** with 50% of maximum yield after 48 h of reaction (63% of substrate consumption), in agreement with the results previously reported for lactose bearing an acetoxy group in anomeric position [21]. By introducing an alkyl or aryl glycoside or thioglycoside group (substrates **11**–**15** in Scheme 3) in anomeric position, the selectivity of AXE moved towards the C2 position allowing the production of compounds **11a**–**15a** with yields ranging from 23% to 52%. When man(1→6)GluNHAc **16** was submitted to hydrolysis, AXE was not able to hydrolyze the acetamido group of the anomeric sugar (GluNAc); thus, the selectivity of the enzyme moved further towards C2′position of the mannose unit at the non-reducing end (Scheme 3). However, the reaction proceeded very slowly (168 h for achieving 40% of substrate consumption) and low yields (about 20% of product **16a**).

**Scheme 4:**
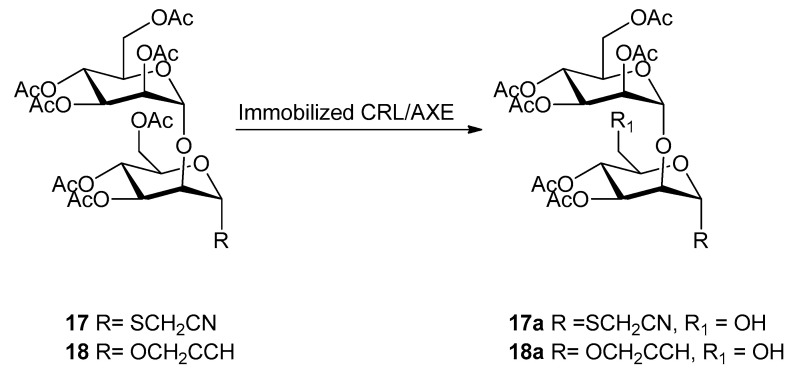
Regioselective enzymatic hydrolysis of peracetylated 1→2 disaccharides (**17**–**18**).

Similarly, when the man(1→2)man disaccharides **17** and **18** were tested as substrates of AXE (Scheme 4), the hydrolysis was observed for the position C6 (being position C2 involved in the glycosidic bond), yielding the products **17a** and **18a** with the thiocyanomethyl and the propargyl group in anomeric position. In these cases, the yields obtained were lower (16% and 20%, respectively) compared to the corresponding man(1→6)man disaccharides (compounds **11a** and **15a**).

## 3. Materials and Methods

Reagents, chemicals, hydrophobic carrier octyl-Sepharose^®^ CL-4B, and lipase from *Candida rugosa* (CRL) were purchased from Sigma-Aldrich (Milano, Italy). Acetyl xylan esterase (AXE) from *Bacillus pumilus* was from Dobfar (Tribiano, Italy). Sepharose^TM^ CL-6B (agarose) was from GE Healthcare (Milan, Italy). α-d-Mannose pentaacetate (**1**) was purchased from Sigma-Aldrich (Milano, Italy).

Compounds purification was performed by flash chromatography using Silica Gel high-purity grade, pore size 60 Å 70–230 mesh, 63–200 μm (Sigma-Aldrich). Analytical thin layer chromatography (TLC) was performed on silica gel F254 precoated aluminium sheets (0.2 mm layer, Merck, Darmstadt, Germany). Products were detected by spraying with 5% H_2_SO_4_ in ethanol, followed by heating to ca. 150 °C. Enzymatic reactions and activity assays were monitored by Titrator 718 stat (pH-Stat) Tritino from Metrohm (Herisau, Switzerland). Characterization of purified compounds was performed by NMR spectroscopy. NMR spectra were recorded in CDCl_3_ on a Bruker Advance III 400 MHz spectrometer (Bruker Corporation, Billerica, MA, USA), available at the Centro Grandi Strumenti of the University of Pavia. All 1D and 2D NMR spectra were acquired using the standard pulse sequences available with Bruker Topspin 3.6 software package. Chemical shifts (δ) are given in ppm and were referenced to the solvent signals (δ_H_ 7.28, δ_C_ 77.00). Signal multiplicities are abbreviated as follows: s, singlet; d, doublet; t, triplet; m, multiplet; b, broad. Structures assignment was performed by means of 2D-COSY and HSQC and, in some cases, 2D-NOESY. Spectra analyses were carried out using Mestrenova reader software. For compounds **5a**, **5b**, **14**, and **18** high resolution mass spectra (HRMS) were recorded with a Bruker Micro-TOF spectrometer in electrospray ionization (ESI) mode, using Tuning-Mix as reference. For all other compounds, mass spectra were recorded on an LCQ-DECA Thermo Finnigan Spectrometer by the ESI (Electron Spray Ionization) ionization method with an ionic source and with use of Xcalibur 2.2 software (Thermo-Finnigan, San Jose, CA, USA). Analyses were run under positive modality, and the experimental conditions were: voltage of the source 5.0 kV, voltage of the capillary 14 V, flow of the gas 35 (arbitrary units), and temperature 200 °C.

### 3.1. Determination of Enzymatic Activity

The activity of the enzymes was determined following a standard protocol by using an automatic titrator pH-Stat. The hydrolytic activity was calculated based on NaOH consumption (mL of NaOH/min).

#### 3.1.1. Standard Activity Assay with Acetyl Xylan Esterase from *Bacillus pumilus* (AXE)

The activity of AXE was determined using 1-naphtyl acetate as standard substrate [21]. The standard reaction mixture was composed of 2 mL of acetonitrile, 2 mL of 1-naphtyl acetate (50 mM in acetonitrile), and 16 mL of phosphate buffer (25 mM, pH 7.0). The reaction was started through the addition of 100 μL soluble enzyme (49 mg/mL) or 10–15 mg of immobilized enzyme. The mixture was mechanically stirred and pH was maintained at 7.0 using 100 mM NaOH as titrant. Experiments were done at least in duplicate.

#### 3.1.2. Standard Activity Assay with *Candida rugosa* Lipase (CRL)

The activity of CRL was determined using tripropionin as standard substrate [36]. The standard reaction mixture was composed of 0.6 mL of acetonitrile, 1 mL of tripropionin, and 18.4 mL of Tris-HCl (25 mM, pH 7.0). The reaction was started through the addition of 100 μL soluble enzyme (10 mg/mL) or 10–15 mg of immobilized enzyme. The mixture was mechanically stirred and pH was maintained at 7.0 using 100 mM NaOH as titrant. Experiments were done at least in duplicate.

### 3.2. AXE Immobilization on Glyoxyl-Agarose (GLX-AG)

GLX-AG was prepared as reported in literature [37]. Briefly, Sepharose^TM^ CL-6B (agarose, 5 g) was suspended in deionized H_2_O (1.4 mL) and NaOH (1.7 M, 2.4 mL) containing NaBH_4_ (28.4 mg/mL). Subsequently, glycidol (1.7 mL) was added dropwise, keeping the vessel at 4 °C in an ice bath. The reaction was kept under gently stirring overnight at 25 °C. After the incubation period, the suspension was filtered, and the carrier was washed abundantly with deionized H_2_O. Oxidation was initiated by adding NaIO_4_ (100 mM, 34 mL). The reaction was carried out for 2 h at room temperature, and then the carrier was filtered under reduced pressure and washed abundantly with deionized H_2_O and stored at 4 °C.

Immobilization of AXE on GLX-AG was performed following a standard protocol [38,39]. Briefly, glyoxyl-agarose was washed abundantly with NaHCO_3_ buffer (50 mM, pH 10) and then filtered under reduced pressure until dryness. Soluble enzyme (50 mg or 150 mg loading of protein per gram of carrier) was solubilized into NaHCO_3_ buffer (50 mM, pH 10). Then, the carrier was added, and the suspension was allowed to stir at 25 °C or 4 °C. Finally, NaBH_4_ (1 mg for each 100 mg of carrier) was added to the mixture and incubated for 30 min to allow imino bonds reduction. The immobilized enzyme was then filtered, rinsed thoroughly with distilled water, and stored at 4 °C till use.

### 3.3. AXE Immobilization on Sepabeads EC-EP/M

Immobilization of AXE on Sepabeads EC-EP/M was performed following a standard protocol [38,39]. Briefly, Sepabeads EC-EP/M was allowed to hydrate for 1 h in water on a rolling shaker at 25 °C, and then it was filtered under reduced pressure until dryness. Soluble enzyme (150 mg loading of protein per gram of carrier) was solubilized into KH_2_PO_4_ buffer (1 M, pH 8). Then, the carrier was added and the suspension was allowed to stir for 24 h at 25 °C. Subsequently, the epoxy groups were quenched with 3 M glycine in KH_2_PO_4_ buffer (1 M, pH 8) for 18 h at 25 °C. The immobilized enzyme was then filtered, rinsed thoroughly with distilled water, and stored at 4 °C till use.

### 3.4. CRL Immobilization on Octyl-Sepharose^®^ (OC-AG)

The crude extract of CRL (1.5 g; loading 2500 UI per gram of carrier) was suspended in KH_2_PO_4_ buffer (25 mM, pH 7.0). The mixture was allowed to stir on the rolling shaker for 30 min. Then, octyl-Sepharose^®^ (3 g), previously conditioned with the same buffer, was added and the suspension was stirred at room temperature overnight. The enzyme derivative was filtered under reduced pressure on a Büchner funnel, rinsed thoroughly with distilled water, and stored at 4 °C till use.

### 3.5. Chemical Synthesis of Monosaccharides ***2**–**9***

Cyanomethyl 2,3,4,6-tetra-*O*-acetyl-1-thio-α-d-mannopyranoside (**2**)

Cyanomethyl 2,3,4,6-tetra*-O-*acetyl-1-thio-α-d-mannopyranoside (**2**) was synthesized as previously reported [40].

Briefly, 2-*S*-(2,3,4,6-tetra*-O-*acetyl-α-d-mannopyranosyl-)-2-thiopseudourea hydrobromide (7.3 g, 0.017 mmol, 1 eq.), sodium meta bisulphite (6.28 g, 0.034 mmol, 2 eq.), and potassium carbonate (2.81 g, 0.0204 mmol, 1.2 eq) were dissolved in acetone/water (50:50, 80 mL). Subsequently, chloroacetonitrile (21.73 mL, 0.34 mmol, 20 eq.) was added, and the reaction was incubated for ~2 h at room temperature. The reaction mixture was monitored by TLC (ethyl acetate/*n*-hexane 5:5, Rf = 0.60). Upon completion, 60 mL of ice water were added to the solution, and the mixture was stirred for 45 min. The reaction was extracted with dichloromethane, and the combined organics extracts were washed with brine, filtered, dried over Na_2_SO_4_, and concentrated in vacuo. The mixture was then crystallized from hot methanol. A white crystalline solid was obtained (3 g, 45%). ^1^H-NMR was in agreement with that previously reported [40].

(4-Methylphenyl) 2,3,4,6-tetra-*O*-acetyl-1-thio-α-d-mannopyranoside (**3**)

(4-Methylphenyl) 2,3,4,6-tetra*-O-*acetyl-1-thio-α-d-mannopyranoside (**3**) was synthesized, slightly modifying the protocol reported by Janssens J. et al. [41].

Briefly, to a mixture of 1,2,3,4,6-penta*-O-*acetyl-α-d-mannopyranose (**1**) (3.372 g, 8.6 mmol, 1 eq.) and *p*-thiocresol (0.955 g, 7.6 mmol, 1.2 eq.) in dichloromethane (35 mL), boron trifluoride diethyl etherate (700 μL, 5.6 mmol, 1.5 eq.) was added dropwise. The mixture was stirred at room temperature for 48 h, under nitrogen atmosphere. The reaction mixture was monitored by TLC (ethyl acetate/toluene 3:7, Rf = 0.57). The reaction mixture was then diluted with dichloromethane (28 mL) and washed with saturated NaHCO_3_ twice and water. The organic layer was dried over MgSO_4_. The solvent was removed, and the residue was purified by column chromatography (SiO_2_, ethyl acetate/toluene 3:7). The desired product was obtained as a white solid (3.21 g, 85%). ^1^H-NMR was in agreement with that previously reported [42].

Ethyl 2,3,4,6-tetra-*O*-acetyl-1-thio-α-d-mannopyranoside (**4**)

Ethyl 2,3,4,6-tetra*-O-*acetyl-1-thio-α-d-mannopyranoside (**4**) was synthesized following a slightly modified protocol reported by Calosso M. et al. [43].

Briefly, to a solution of 1,2,3,4,6-penta*-O-*acetyl-α-d-mannopyranose (**1**) (3.062 g, 7.85 mmol, 1 eq.) in anhydrous dichloromethane, ethanethiol (0.79 mL, 11 mmol, 1.4 eq.) in the presence of 4 Å molecular sieves was added. The reaction was cooled to 0 °C, and BF_3_OEt_2_ (1.65 mL, 13.345 mmol, 1.7 eq.) was added dropwise. The reaction was monitored by TLC (ethyl acetate/*n*-hexane 6:4, Rf = 0.64). After 7 h, the reaction was washed with 40 mL of a saturated solution of NaHCO_3_, and the aqueous phase was washed with dichloromethane. The organic phase was dried with Na_2_SO_4_, filtered and concentrated in vacuo. The reaction crude was purified by flash chromatography (SiO_2_, ethyl acetate/*n*-hexane 6:4). The desired product was obtained as a white solid (2.15 g, 70%). ^1^H-NMR was in agreement with that previously reported [44].

Propargyl 2,3,4,6-tetra-*O*-acetyl-α-d-mannopyranoside (**5**)

Propargyl 2,3,4,6-tetra*-O-*acetyl-α-d-mannopyranoside (**5**) was synthesized following a standard procedure [45]. Briefly, 1,2,3,4,6-penta*-O-*acetyl-α-d-mannopyranose (**1**) (1.043 g, 2.68 mmol, 1 eq.) was dissolved in anhydrous dichloromethane (8 mL) under nitrogen atmosphere in presence of activated molecular sieves. Propargyl alcohol (0.156 mL, 2.68 mmol, 1 eq.) was added. The mixture was cooled to 0 °C, and BF_3_OEt_2_ (0.661 mL, 5.36 mmol, 2 eq.) was added dropwise. The reaction mixture was allowed to warm up to room temperature and allowed to stir for 5 days. The solution was diluted with dichloromethane, washed with saturated NaHCO_3_ then water, dried over Na_2_SO_4_, and concentrated in vacuo. The reaction was monitored by TLC (ethyl acetate/*n*-hexane 5:5, Rf = 0.30). Column chromatography (SiO_2_, ethyl acetate/*n*-hexane 5:5) gave the desired compound as white solid (2.64 g, 84%). ^1^H-NMR was in agreement with that previously reported [45].

Allyl 2,3,4,6-tetra-*O*-acetyl-α-d-mannopyranoside (**6**)

Allyl 2,3,4,6-tetra*-O-*acetyl-α-d-mannopyranoside (**6**) was synthesized modifying slightly the protocol reported by Balcerzak A.K. et al. [46]. Briefly, 1,2,3,4,6-penta*-O-*acetyl-α-d-mannopyranose (**1**) (1 g, 25.6 mmol, 1 eq.) was dissolved in anhydrous dichloromethane (8 mL) under nitrogen atmosphere in presence of activated molecular sieves. Allyl alcohol (0.175 mL, 25.6 mmol, 1 eq.) was added. The mixture was cooled to 0 °C, and BF_3_OEt_2_ (0.633 mL, 51.2 mmol, 2 eq.) was added dropwise. The reaction mixture was allowed to warm up to room temperature and allowed to stir for 7 days. The solution was diluted with dichloromethane, washed with saturated NaHCO_3_ and then water, dried over Na_2_SO_4_, and concentrated in vacuo. The reaction was monitored by TLC (ethyl acetate/*n*-hexane 5:5, Rf = 0.65). Column chromatography (SiO_2_, ethyl acetate/*n*-hexane 5:5) gave the desired product as a colorless oil (610 mg, 61%). ^1^H-NMR was in agreement with that previously reported [47].

(3-Azidopropyl) 2,3,4,6-tetra-*O*-acetate-α-d-mannopyranoside (**7**)

(3-Azidopropyl) 2,3,4,6-tetra*-O-*acetate-α-d-mannopyranoside (**7**) was synthesized as previously reported [34]. Briefly, 3-azido-1-propanol (0.31 mL, 3.08 mmol) and BF_3_·Et_2_O (0.49 mL, 3.87 mmol) were added to a solution of 1,2,3,4,6-penta*-O-*acetyl-α-d-mannopyranose (**1**) (1.0 g, 2.54 mmol) in dichloromethane (20 mL) at 0 °C, and the mixture was stirred at room temperature overnight. The reaction was monitored by TLC (ethyl acetate/*n*-hexane 5:5, Rf = 0.25). The reaction mixture was quenched by adding dichloromethane (10 mL) and saturated NaHCO_3_ to neutralize the remaining BF_3_Et_2_O. The aqueous layer was extracted by dichloromethane (30 mL), and the combined organic layers were washed with brine and then dried over anhydrous MgSO_4_, affording the crude product. The crude product was purified by flash chromatography (SiO_2_, ethyl acetate/*n*-hexane 1:1) to yield the desired product as colorless oil (0.58 g, 50%). ^1^H-NMR was in agreement with that previously reported [48].

Cyanomethyl 2-acetamido-3,4,6-tri-*O*-acetyl-2-deoxy-1-thio-β-d-glucopyranoside (**8**)

Cyanomethyl 2-acetamido-3,4,6-tri*-O-*acetyl-2-deoxy-1-thio-α-d-glucopyranoside (**8**) was synthesized as reported by Zheng C. et al. [15]. Briefly, 1-thiourea-2-acetamido-3,4,6,-tri*-O-*acetyl-2-deoxy-α-d-glucopyranoside (245 mg, 0.554 mmol) was dissolved in 1:1 water:acetone mixture (2.6 mL), and sodium meta bisulphite (0.212 g, 1.115 mmol), potassium carbonate (0.093 g, 0.672 mmol) and chloroacetonitrile (0.712 mL, 20 eq.) were added. The mixture was stirred at room temperature, and reaction was monitored by TLC (dichloromethane/methanol 9:1, Rf = 0.64). Upon completion, 8 mL of ice water were added to the solution that was stirred for 45 min. The reaction was extracted with dichloromethane, and the combined organics extracts were washed with brine and dried over anhydrous Na_2_SO_4_ and concentrated in vacuo. The reaction crude was purified by flash chromatography (SiO_2_, dichloromethane/methanol 95:5). The desired product was obtained as a white solid (230 mg, 98%). ^1^H-NMR was in agreement with that previously reported [15].

2,3,4,6-Tetra-*O*-acetyl-α-d-mannopyranosyl trichloracetimidate (**9**)

2,3,4,6-Tetra*-O-*acetyl-α-d-mannopyranosyl trichloracetimidate (**9**) was synthesized following the procedure reported by Ekholm F.S. et al. [49]. Briefly, trichloroacetonitrile (4.97 mL, 49.55 mmol, 5 eq.) and 1,8-diazabicyclo-[5,4,0]-7-undecene (DBU, 0.74 mL, 4.95 mmol, 0.5 eq.) were added to a solution of 2,3,4,6-tetra*-O-*acetyl-d-mannopyranose (**1b**) (3.45 g, 9.91 mmol, 1 eq.) in anhydrous dichloromethane (30 mL) at 0 °C under nitrogen atmosphere. The mixture was stirred for 3 h at 0 °C and then concentrated in vacuo. The reaction mixture was monitored by TLC (ethyl acetate/*n*-hexane 5:5, Rf = 0.8) and purified by flash chromatography (SiO_2_, ethyl acetate/*n*-hexane 5:5). The desired product was obtained as a yellow sticky solid (3.89 g, 80%). ^1^H-NMR was in agreement with that previously reported [49].

### 3.6. Enzymatic Deprotection of Monosaccharides ***1**–**8***

The deacetylated monosaccharides were produced following a general procedure of hydrolysis.

The substrates (10 mM final concentration) were dissolved in acetonitrile (20%–30% *v*/*v* depending on substrate solubility) under magnetic stirring, and then phosphate buffer (50 mM, pH 4.0–5.0) was added slowly. The reaction was started through the addition of immobilized CRL and/or AXE, previously conditioned with reaction buffer. The reactions were performed at 25 °C under mechanical stirring; the pH of the solution was maintained constant by automatic titration. Reaction course was monitored by TLC.

After complete consumption of the starting substrate or before an excessive formation of undesired products, the reactions were stopped by enzymatic derivative filtration on Büchner funnel. Acetonitrile was evaporated under reduced pressure, and the solution was brined and extracted with ethyl acetate. The organic layers were dried over anhydrous Na_2_SO_4_, filtered, and concentrated in vacuo. The mixture obtained was purified by flash chromatography.

1,2,3,4-Tetra-*O*-acetyl-α-d-mannopyranose (**1a**)

1,2,3,4,6-Penta*-O-*acetyl-α-d-mannopyranose (**1**) was hydrolyzed to the corresponding 1,2,3,4-tetra*-O-*acetyl-α-d-mannopyranose (**1a**) by CRL-OC-AG as reported by Bavaro T. et al. [17]. ^1^H-NMR was in agreement with that previously reported [50].

2,3,4,6-Tetra-*O*-acetyl-α-d-mannopyranose (**1b**)

1,2,3,4,6-Penta*-O-*acetyl-α-d-mannopyranose (**1**) (30 mg, 5mM) was hydrolyzed to the corresponding 2,3,4,6-tetra*-O-*acetyl-α-d-mannopyranose (**1b**) by AXE-GLX-AG. ^1^H-NMR was in agreement with that previously reported [49].

Cyanomethyl 2,3,4-tri-*O*-acetyl-1-thio-α-d-mannopyranoside (**2a**)

Cyanomethyl 2,3,4,6-tetra*-O-*acetyl-1-thio-α-d-mannopyranoside (**2**) was hydrolyzed to the corresponding cyanomethyl-2,3,4-tri*-O-*acetyl-1-thio-α-d-mannopyranoside (**2a**) by CRL-OC-AG as reported by Bavaro T. et al. [17].

^1^H-NMR was in agreement with that previously reported [17].

Cyanomethyl 3,4,6-tri-*O*-acetyl-1-thio-α-d-mannopyranoside (**2b**)

Cyanomethyl 2,3,4,6-tetra*-O-*acetyl-1-thio-α-d-mannopyranoside (**2**) was hydrolyzed to the corresponding cyanomethyl 3,4,6-tri*-O-*acetyl-1-thio-α-d-mannopyranoside (**2b**) by CRL-OC-AG as reported by Bavaro T. et al. [17].

^1^H-NMR was in agreement with that previously reported [17].

(4-Methylphenyl) 2,3,4-tri-*O*-acetyl-1-thio-α-d-mannopyranoside (**3a**)

(4-Methylphenyl) 2,3,4,6-tetra*-O-*acetyl-1-thio-α-d-mannopyranoside (**3**) was hydrolyzed to the corresponding (4-methylphenyl) 2,3,4-tri*-O-*acetyl-1-thio-α-d-mannopyranoside (**3a**) following the general procedure for enzymatic hydrolysis: substrate (1g, 10 mM) was solubilized in 255 mL of phosphate buffer 50 mM pH 4.0 and 30% *v*/*v* of acetonitrile. The reaction was started through addition of CRL-OC-AG (7000 UI). The reaction mixture was monitored by TLC (ethyl acetate/*n*-hexane 6:4) and purified by flash chromatography (SiO_2_, ethyl acetate/*n*-hexane 6:4, Rf = 0,56). The desired product was obtained as a white solid. (590 mg, 65%).

^1^H-NMR (400 MHz, CDCl_3_): δ 7.38 (d, 2H, *J* = 8.0 Hz, Ar), 7.12 (d, 2H, *J* = 8.0 Hz, Ar), 5.50 (s, 1H), 5.42 (s, 1H), 5.30-5.35 (m, 2H), 4.30 (m, 1H), 3.7 (m, 2H), 2.32 (s, 3H, Ph-*C*H_3_), 2.13, 2.09, 2.02 (3s, 9H, OAc).

^13^C-NMR (300 MHz, CDCl_3_): 170.74, 169.97, 169.82 (*C*OOCH_3_), 138.49, 132.67, 130.04, 128.83 (Ar), 86.12, 71.74, 70.98, 69.21, 66.62, 61.30, 21.12 (Ph-*C*H_3_), 20.87, 20.75, 20.66 (COO*C*H_3_).

MS: *m*/*z* = 435.13 [M + Na^+^] (calculated 435.45).

Ethyl 2,3,4-tri-*O*-acetyl-1-thio-α-d-mannopyranoside (**4a**)

Ethyl 2,3,4,6-tetra*-O-*acetyl-1-thio-α-d-mannopyranoside (**4**) was hydrolyzed to the corresponding ethyl 2,3,4-tri*-O-*acetyl-1-thio-α-d-mannopyranoside (**4a**) following the general procedure for enzymatic hydrolysis: substrate (120 mg, 10 mM) was solubilized in 30.6 mL of phosphate buffer 50 mM pH 4.0 and 30% *v*/*v* of acetonitrile. The reaction was started through addition of CRL-OC-AG (500 UI). The reaction mixture was monitored by TLC (ethyl acetate/*n*-hexane 6:4) and purified by flash chromatography (SiO_2_, ethyl acetate/*n*-hexane 6:4, Rf = 0.46). The desired product was obtained as a colorless oil (56.8 mg, 53%).

^1^H-NMR (400 Hz, CDCl_3_): δ 5.36-5.28 (m, 4H, H-1, H-2, H-3, H-4), 4.16-4.14 (m, 1H, H-5), 3.70- 3.67 (m, 2H, H-6_ab_), 2.67-2.62 (m, 2H, SC*H_2_*CH_3_), 2.09, 2.02, 1.93 (3s, 9H, OAc), 1.30 (t, 3H, SCH_2_C*H_3_*).

^13^C-NMR (400 Hz, CDCl_3_): δ 170.93, 170.07, 169.79 (*C*OOCH_3_), 82.15 (C-1), 71.27, 71.06, 69.27, 66.69, 61.23 (5C, ring carbon), 25.39 (S*C*H_2_CH_3_), 20.91, 20.74, 20.65 (COO*C*H_3_), 14.68 (SCH_2_*C*H_3_).

MS: *m*/*z* = 373.10 [M + Na^+^] (calculated 373.38).

Propargyl 2,3,4-tri-*O*-acetyl-α-d-mannopyranoside (**5a**)

Propargyl 2,3,4,6-tetra*-O-*acetyl-α-d-mannopyranoside (**5**) was hydrolyzed to the corresponding propargyl 2,3,4-tri*-O-*acetyl-α-d-mannopyranoside (**5a**) following the general procedure for enzymatic hydrolysis: substrate (500g, 10 mM) was solubilized in 255 mL of phosphate buffer 50 mM pH 4.0 and 30% *v*/*v* of acetonitrile. The reaction was started through addition of CRL-OC-AG (7000 UI). The reaction mixture was monitored by TLC (ethyl acetate/*n*-hexane 6:4, Rf = 0.37) and purified by flash chromatography (SiO_2_, ethyl acetate/*n*-hexane 5:5). The desired product was obtained as a white solid (311 mg, 80%).

^1^H-NMR (400 MHz, CDCl_3_): δ 5.33 (dd, 1H, *J* = 3.4, 10.3 Hz, H-3), 5.27-5.16 (m, 2H, H-2, H-4), 4.98 (s, 1H, H-1), 4.21 (d, 2H, *J* = 2.4 Hz, OC*H_2_*C≡CH), 3.78-3.72 (m, 1H, H-5), 3.65 (dd, 1H, *J* = 2.4, 12.7 Hz, H-6_a_), 3.56 (dd, 1H, *J* = 4.23, 12.7 Hz, H-6_b_), 2.41 (t, 1H, *J* = 2.4 Hz, C≡C*H*), 2.17-2.09-2.02 (3s, 9H, CH_3_COO).

^13^C-NMR (400 MHz, CDCl_3_): δ 170.76, 169,97, 169.80 (3C, CH_3_*C*OO), 96.34 (C-1), 78.06 (CH≡*C*-), 75.50 (*C*H≡C-), 71.14, 69.42, 68.72, 66.34, 61.16, 54.96(CH≡C*C*H_2_), 20.84, 20.72, 20.66 (3C, *C*H_3_COO).

HRMS: *m*/*z* = 367.0996 [M + Na^+^] (calculated 367.100).

Propargyl 3,4,6-tri-*O*-acetyl-α-d-mannopyranoside (**5b**)

Propargyl 2,3,4,6-tetra*-O-*acetyl-α-d-mannopyranoside (**5**) was hydrolyzed to the corresponding propargyl 3,4,6-tri*-O-*acetyl-α-d-mannopyranoside (**5b**) following the general procedure for enzymatic hydrolysis: substrate (500 mg, 10 mM) was solubilized in 234 mL of phosphate buffer 50 mM pH 4.0 and 30% *v/v* of acetonitrile. The reaction was started through addition of immobilized AXE (787.5 UI). The reaction mixture was monitored by TLC (ethyl acetate/*n*-hexane 6:4, Rf = 0.41) and purified by flash chromatography (SiO_2_, ethyl acetate/*n*-hexane 6:4). The desired product was obtained as a white sticky solid (178 mg, 40%).

^1^H-NMR (300 MHz, CDCl_3_): δ 5.29 (t, 1H, *J* = 9.9 Hz, H-4), 5.17 (dd, 1H, *J* = 9.9, 3.2 Hz, H-3), 5.01 (d, 1H, *J* = 1.8 Hz, H-1), 4-26-4.15 (m, 3H, H-6_a_, OC*H_2_*C≡CH), 4.06 (d, 1H, *J* = 2.5 Hz H-6), 4.02 (m, 1H, H-2), 3.90 (m, 1H, H-5), 2.43 (t, 1H, *J* = 2.5 Hz, C≡C*H*), 2.02, 2.01, 1.97 (3s, 9H, COCH_3_).

^13^C-NMR (300 MHz, CDCl_3_): δ 170.85, 169.99, 169.89 (3C, CH_3_*C*OO), 98.16 (C-1), 78.30 (CH≡*C*-), 75.33 (*C*H≡C-), 71.53 (C-3), 69.02, 68.83 (C-2 C-5), 66.12 (C-4), 62.35 (C-6), 54.77 (CH≡C-*C*H_2_-O), 20.83, 20.73, 20.67 (3C, *C*H_3_COO).

HRMS: *m*/*z* = 367.100 [M + Na^+^] (calculated 367.100).

Allyl 2,3,4-tri-*O*-acetyl-α-d-mannopyranoside (**6a**)

Allyl 2,3,4,6-tetra*-O-*acetyl-α-d-mannopyranoside (**6**) was hydrolyzed to the corresponding allyl 2,3,4-tri*-O-*acetyl-α-d-mannopyranoside (**6a**) following the general procedure for enzymatic hydrolysis: substrate (300 mg, 10 mM) was solubilized in 77.3 mL of phosphate buffer 50 mM pH 4.0 and 30% *v*/*v* of acetonitrile. The reaction was started through addition of CRL-OC-AG (4372.5 UI). The reaction mixture was monitored by TLC (ethyl acetate/*n*-hexane 5:5, Rf = 0.4) and purified by flash chromatography (SiO_2_, ethyl acetate/*n*-hexane 5:5). The desired product was obtained as a colorless oil (214 mg, 80%). ^1^H-NMR was in agreement with that previously reported [51].

(3-Azidopropyl) 2,3,4-tri-*O*-acetate-α-d-mannopyranoside (**7a**)

(3-Azidopropyl) 2,3,4,6-tetra*-O-*acetate-α-d-mannopyranoside (**7**) was hydrolyzed to the corresponding (3-azidopropyl) 2,3,4-tri*-O-*acetate-α-d-mannopyranoside (**7a**) by CRL-OC-AG as reported by Li Z. et al. [34]. ^1^H-NMR was in agreement with those previously reported [34].

Cyanomethyl 2-acetamido-3,4-di-*O*-acetyl-2-deoxy-1-thio-β-d-glucopyranoside (**8a**)

Cyanomethyl 2-acetamido-3,4,6-tri*-O-*acetyl-β-d-glucopyranoside (**8**) was hydrolyzed to the corresponding 1-thiocyanomethyl-2-acetamido-3,4-di*-O-*acetyl-β-d-glucopyranoside (**8a**) by CRL-OC-AG as reported by Zheng C. et al. [15]. ^1^H-NMR was in agreement with that previously reported [15].

### 3.7. Chemical Synthesis of Disaccharides ***10**–**18***

2′,3′,4′,6′-Tetra-*O*-acetyl-α-d-mannopyranosyl-(1→6)-1,2,3,4-tetra-*O*-acetyl-α-d-mannopyranose (**10**)

2,3,4,6-Tetra*-O-*acetyl-α-d-mannopyranosyl trichloracetimidate (**9**) (3.77 g, 7.662 mmol, 1.6 eq.) and 1,2,3,4-tetra*-O-*acetyl-α-d-mannopyranose (**1a**) (1.67 g, 4.798 mmol, 1 eq.) were dissolved in dry dichloromethane (50 mL) in presence of activated molecular sieves and cooled to 0 °C under nitrogen atmosphere. BF_3_OEt_2_ (591 μL, 4.798 mmol, 1 eq.) was added, and the mixture was stirred at room temperature for 2.5 h. The reaction was quenched with triethylamine (668 μL, 4.798 mmol, 2 eq.), stirred for 5 min, filtered, and concentrated in vacuo. The reaction mixture was monitored by TLC (dichloromethane/methanol 9:1, Rf = 0.89) and purified by flash chromatography (SiO_2_, dichloromethane/methanol 9:1). The desired product was obtained as a white solid (2.83 g, 87%). ^1^H-NMR was in agreement with that previously reported [52].

Cyanomethyl (2′,3′,4′,6′-tetra-*O*-acetyl-α-d-mannopyranosyl)-(1→6)-2,3,4-tri-*O*-acetyl-1-thio-α-d-mannopyranoside (**11**)

Cyanomethyl (2′,3′,4′,6′-tetra*-O-*acetyl-α-d-mannopyranosyl)-(1→6)-2,3,4-tri*-O-*acetyl-1-thio-

α-d-mannopyranoside (**11**) was synthesized as reported by Bavaro T. et al. [17].

Briefly, 2,3,4,6-tetra*-O-*acetyl-α-d-mannopyranosyl trichloracetimidate (**9**) (226 mg, 0.460 mmol, 2 eq.) and cyanomethyl 3,4,6-tri*-O-*acetyl-1-thio-α-d-mannopyranoside (**2a**) (83 mg, 0.230 mmol, 1 eq.) were dissolved in dry dichloromethane (5 mL) in presence of activated molecular sieves and cooled to −70 °C with dry ice under nitrogen atmosphere. BF_3_OEt_2_ (56.6 μL, 0.460 mmol, 2 eq.) was added, and the mixture was stirred at room temperature for 4 h. The reaction was quenched with triethylamine (64.2 μL, 0.460 mmol, 2 eq.), stirred for 5 min, filtered, and concentrated in vacuo. The reaction mixture was monitored by TLC (ethyl acetate/*n*-hexane 5:5, Rf = 0.32) and purified by flash chromatography (SiO_2_, ethyl acetate/*n*-hexane 5:5). The desired product was obtained as a white solid (125 mg, 79%). ^1^H-NMR was in agreement with that previously reported [17].

(4-Methylphenyl)(2′,3′,4′,6′-tetra-*O*-acetyl-α-d-mannopyranosyl)-(1→6)-2,3,4-tri-*O*-acetyl-1-thio-α-d-mannopyranoside (**12**)

2,3,4,6-Tetra*-O-*acetyl-α-d-mannopyranosyl trichloracetimidate (**9**) (366 mg, 0.743 mmol, 2.5 eq.) and (4-methylphenyl) 2,3,4-tri*-O-*acetyl-1-thio-α-d-mannopyranoside (**3a**) (140 mg, 0.298 mmol, 1 eq.) were dissolved in dry dichloromethane (7 mL) in presence of 4 Å molecular sieves and cooled to 0 °C under nitrogen atmosphere. BF_3_OEt_2_ (73.5 μL, 0.596 mmol, 2 eq.) was added, and the mixture was stirred at 0 °C for 3.5 h. The reaction was quenched with triethylamine (83.2 μL, 0.596 mmol, 2 eq.), stirred for 5 min, filtered, and concentrated in vacuo. The reaction mixture was monitored by TLC (ethyl acetate/*n*-hexane 5:5, Rf = 0.28) and purified by flash chromatography (SiO_2_, ethyl acetate/*n*-hexane 5:5). The desired product was obtained as a white solid (204 mg, 92%).

^1^H-NMR (400 MHz, CDCl_3_): δ 7.30 (d, 2H *J* = 8.0 Hz, Ar), 7.08 (d, 2H, *J* = 8.0 Hz, Ar), 5.50 (dd, *J* = 1.7, 3.2 Hz, 1H, H-3), 5.42–5.20 (m, 6H, H-4, H-2′, H-3′, H-2, H-1, H-4′), 4.84 (d, 1H, *J* = 1.5 Hz, H-1′), 4.43–4.38 (m, 1H, H-5), 4.21 (dd, 1H, *J* = 5.0, 12.2 Hz, H-6′_a_), 3.99 (dd, 1H, *J* = 12.3, 2.4 Hz, H-6′_b_), 3.95–3.88 (m, 1H, H-5′), 3.74 (dd, 1H, *J* = 11.3, 5.0 Hz, H-6_a_), 3.59 (dd, 1H, *J* = 11.3, 2.7 Hz, H-6_b_), 2.25 (s, 3H, C*H_3_*-Ar), 2.08, 2.09, 2.02, 2.02, 1.99, 1.95, 1.92 (7s, 21H, COC*H_3_*).

^13^C-NMR (400 MHz, CDCl_3_): δ 170.60, 170.14, 169.90, 169.87, 169.74, 169.72, 169.56 (7C, CH_3_*C*OO), 138.34, 132.58, 130.08, 129.08 (Ar), 97.98 (C-1′), 86.17 (C-1), 70.91 (C-3), 70.22 (C-5), 69.44, 69.31, 69.07, 68.62 (C-5′), 66.85 (C-6), 66.65, 66.00, 62.31 (C-6′), 21.13(CH_3_-Ar), 20.85, 20.78, 20.75, 20.72, 20.72, 20.66, 20.62 (7C, *C*H_3_COO).

MS: *m*/*z* = 765.18 [M + Na^+^] (calculated 765.20).

Ethyl (2′,3′,4′,6′-tetra-*O*-acetyl-α-d-mannopyranosyl)-(1→6)-2,3,4-tri-*O*-acetyl-1-thio-α-d-mannopyranoside (**13**)

2,3,4,6-Tetra*-O-*acetyl-α-d-mannopyranosyl trichloracetimidate (**9**) (58.7 mg, 0.119 mmol, 1 eq.) and ethyl 2,3,4-tri*-O-*acetyl-1-thio-α-d-mannopyranoside (**4a**) (41.8 mg, 0.119 mmol, 1 eq.) were dissolved in dry dichloromethane (1 mL) in presence of 4 Å molecular sieves and cooled to 0 °C under nitrogen atmosphere. BF_3_OEt_2_ (14.7 μL, 0.119 mmol, 1 eq.) was added, and the mixture was stirred at room temperature for 4 h. The reaction was quenched with triethylamine (17 μL, 0.119 mmol, 1 eq.), stirred for 5 min, filtered, and concentrated in vacuo. The reaction mixture was monitored by TLC (dichloromethane/acetone 9:1, Rf = 0.71) and purified by flash chromatography (SiO_2_, dichloromethane/acetone 9:1). The desired product was obtained as a white solid (28 mg, 37%).

^1^H-NMR (400 MHz, CDCl_3_): δ 5.38–5.22 (m, 7H, H-2′, H-3′, H-4′, H-1, H-2, H-3, H-4), 4.85 (d, 1H, *J* = 1.7 Hz, H-1′), 4.39 (ddd, 1H, *J* = 9.1, 6.4, 2.3 Hz, H-5), 4.25 (dd, 1H, *J* = 12.2, 5.4 Hz, H-6′_a_), 4.16 (dd, 1H, *J* = 12.2, 2.4 Hz, H-6′_b_), 4.06 (ddd, 1H, *J* = 9.5, 5.4, 2.4 Hz, 1H, H-5′), 3.82 (dd, 1H, *J* = 10.9, 6.4 Hz, H-6_a_), 3.56 (dd, 1H, *J* = 10.8, 2.4 Hz, H-6_b_), 2.74–2.63 (m, 2H, SC*H*_2_CH_3_), 2.18, 2.17, 2.13, 2.08, 2.05, 2.00, 1.99 (7s, 21H, COC*H_3_*), 1.34 (t, 3H, *J* = 7.4, SCH_2_C*H_3_*).

^13^C-NMR (400 MHz, CDCl_3_): δ 170.61, 170.05, 169.99, 169.88, 169.77, 169.74, 169.66 (7C, CH_3_*C*OO), 97.33 (C-1′), 81.47 (C-1), 71.04, 69.56, 69.54 (C-5), 69.41, 68.88, 68.59 (C-5′), 66.81 (C-6), 66.53, 66.05, 62.39 (C-6′), 25.08 (S*C*H_2_CH_3_), 20.86, 20.84, 20.74, 20.74, 20.69, 20.63, 20.63 (7C, *C*H_3_COO), 14.57 (SCH_2_*C*H_3_).

MS: *m*/*z* = 703.12 [M + Na^+^] (calculated 703.19).

Propargyl (2′,3′,4′,6′-tetra-*O*-acetyl-α-d-mannopyranosyl)-(1→6)-2,3,4-tri-*O*-acetyl-α-d-mannopyranoside (**14**)

2,3,4,6-Tetra*-O-*acetyl-α-d-mannopyranosyl trichloracetimidate (**9**) (1.1 g, 2.233 mmol, 2 eq.) and propargyl 2,3,4-tri*-O-*acetyl-α-d-mannopyranoside (**5a**) (0.384 g, 1.115 mmol, 1 eq.) were dissolved in dry dichloromethane (35 mL) in presence of 4 Å molecular sieves and cooled to 0 °C under argon atmosphere. BF_3_OEt_2_ (137.6 µL, 1.115 mmol, 1 eq.) was added, and the mixture was stirred at 0 °C for 4 h. The reaction was quenched with triethylamine (155 µL, 1.115 mmol, 1 eq.), stirred for 5 min, filtered over Celite, and concentrated in vacuo. The reaction mixture was monitored by TLC (dichloromethane/acetone 9:1, Rf = 0.61). Column chromatography (SiO_2_, dichloromethane/acetone 9:1) gave the desired product as a white solid (0.676 g, 90%).

^1^H-NMR (400 MHz, CDCl_3_): δ 5.38-5.25 (m, 6H, H-4′, H-3′, H-2′, H-4, H-3, H-2), 5.03 (d, 1H, *J* = 1.8 Hz, H-1), 4.87 (d, 1H, *J* = 1.7 Hz, H-1′), 4.32 (d, 2H, *J* = 2.4 Hz, OC*H_2_*C≡CH), 4.28 (dd, 1H, *J* = 5.3 Hz, 12.4 Hz, H-6′_a_), 4.15 (dd, 1H, *J* = 2.4 Hz, 12.2 Hz, H-6′_b_), 4.12-3.98 (m, 2H, H-5′, H-5), 3.80 (dd, 1H, *J* = 5.7 Hz, 11 Hz, H-6_b_), 3.60 (dd, 1H, *J* = 2.6 Hz, 11 Hz, H-6_a_), 2.53 (t, 1H, *J* = 2.4 Hz, C≡C*H*), 2.18, 2.17, 2.12, 2.07, 2.06, 2.01, 2.00 (7s, 21H, COCH_3_).

^13^C-NMR (600 MHz, CDCl_3_): δ 170.59, 170.04, 169.94, 169.83, 169.79, 169.74, 169.70 (7C, CH_3_*C*OO), 97.51 (C-1′), 96.03 (C-1), 78.05 (OCH_2_*C*≡CH), 75.60 (OCH_2_C≡*C*H), 69.85(C-5), 69.35, 69.29, 69.01, 68.98, 68.65 (C-5′), 66.64 (C-6), 66.44, 65.97, 62.42 (C-6′), 54.95 (O*C*H_2_C≡CH), 20.84, 20.75, 20.72, 20.70, 20.70, 20.64, 20.62 (7C, *C*H_3_COO).

HRMS: *m*/*z* = 697.1943 [M + Na^+^] (calculated 697.1950).

Allyl (2′,3′,4′,6′-tetra-*O*-acetyl-α-d-mannopyranosyl)-(1→6)-2,3,4-tri-*O*-acetyl-α-d-mannopyranoside (**15**)

2,3,4,6-Tetra*-O-*acetyl-α-d-mannopyranosyl trichloracetimidate (**9**) (226.4 mg, 0.460 mmol, 1 eq.) and allyl 2,3,4-tri*-O-*acetyl-α-d-mannopyranoside (**6a**) (159 mg, 0.460 mmol, 1 eq.) were dissolved in dry dichloromethane (5 mL) in presence of activated molecular sieves and cooled to 0 °C under nitrogen atmosphere. BF_3_OEt_2_ (56 μL, 0.460 mmol, 1 eq.) was added, and the mixture was stirred at 0 °C for 2 h. The reaction was quenched with triethylamine (64.2 μL, 0.460 mmol, 1 eq.), stirred for 5 min, filtered, and concentrated in vacuo. The reaction mixture was monitored by TLC (dichloromethane/acetone 9:1, Rf = 0.74) and purified by flash chromatography (SiO_2_, toluene/methanol 9:1). The desired product was obtained as white solid (156 mg, 50%).

^1^H-NMR (400 MHz, CDCl_3_): δ 5.98-5.88 (m, 1H, CH_2_C*H*=CH_2_), 5.41-5.23 (m, 8H, H-2, H-3, H-4 H-2′, H-3′, H-4′, CH_2_CH=C*H_2_*), 4.88 (d, 1H, *J* = 1.8 Hz, H-1), 4.86 (d, 1H, *J* = 1.8 Hz, H-1′), 4.30-4.21 (m, 2H, H-6′_a_, C*H_2_*CH=CH_2_), 4.16 (dd, 1H, *J* = 12.2, 2.4 Hz, H-5), 4.14-3.97 (m, 3H, H-5′, H-6′_b_, C*H_2_*CH=CH_2_), 3.80 (dd, 1H, *J* = 10.9, 6.0 Hz, H-6_a_), 3.58 (dd, 1H, *J* = 10.9, 2.5 Hz, H-6_b_), 2.17, 2.17, 2.13, 2.07, 2.06, 2.01, 2.00 (7s, 21H, C*H_3_*COO).

^13^C-NMR (400 MHz, CDCl_3_): δ 170.63, 170.18, 169.99, 169.89, 169.89, 169.77, 169.69 (7C, COO), 133.04 (CH_2_*C*H=CH_2_), 118.42 (CH_2_CH=*C*H_2_), 97.43 (C-1), 96.26 (C-1′), 69.61, 69.42, 69.36, 69.17, 68.95, 68.64, 68.55 (*CH_2_*CH=CH_2_), 66.63, 66.63, 66.04 (C-6), 62.42 (C-6′), 20.87, 20.82, 20.74, 20.74, 20.70, 20.70, 20.64 (7C, *C*H_3_COO).

MS: *m*/*z* = 699.25 [M + Na^+^] (calculated 699.21).

Cyanomethyl (2′,3′,4′,6′-tetra-*O*-acetyl-α-d-mannopyranosyl)-(1→6)-2-acetamido-3,4-di-*O*-acetyl-2-deoxy-1-thio-β-d-glucopyranoside (**16**)

Cyanomethyl (2′,3′,4′,6′-tetra*-O-*acetyl-α-d-mannopyranosyl)-(1→6)-2-acetamido-3,4-di*-O-*acetyl-2-deoxy-1-thio-β-d-glucopyranoside (**16**) was synthesized as reported by Zheng C. et al. [15]. Briefly, 2,3,4,6-tetra*-O-*acetyl-α-d-mannopyranosyl trichloracetimidate (**9**) (235 mg, 0.478 mmol, 2 eq.) and cyanomethyl 2-acetamido-3,4-di*-O-*acetyl-2-deoxy-1-thio-β-d-glucopyranoside (**8a**) (86 mg, 0.239 mmol, 1 eq.) were dissolved in dry dichloromethane (25 mL) in presence of activated molecular sieves and cooled to 0 °C under nitrogen atmosphere. BF_3_OEt_2_ (59 μL, 0.478 mmol, 2 eq.) was added, and the mixture was stirred at room temperature for 2.5 h. The reaction was quenched with triethylamine (67 μL, 0.478 mmol, 2 eq.), stirred for 5 min, filtered, and concentrated in vacuo. The reaction mixture was monitored by TLC (ethyl acetate/diethyl ether 3:2, Rf = 0.39) and purified by flash chromatography (SiO_2_, ethyl acetate/diethyl ether 3:2). The desired product was obtained as a as a colorless oil (122 mg, 74%).

^1^H-NMR (400 MHz, CDCl_3_): δ 5.95 (d, 1H, *J* = 9.2 Hz, NH), 5.39–5.17 (m, 4H, H-4′, H-3′, H-2′, H-3), 5.05 (t, 1H, *J* = 9.3 Hz, H-4), 4.86 (s, 1H, H-1′), 4.80 (d, 1H, *J* = 10.3 Hz, H-1), 4.29 (dd, 1H, *J* = 12.4, 5.1 Hz, H-6′_a_), 4.25-4.09 (m, 2H, H-2, H-6′_b_), 4.02 (m, broad, 1H, H-5′), 3.83-3.76 (m, 2H, H-6_a_, H-5), 3.73 (d, 1H, *J* = 17.1 Hz, C*H*CN) 3.63-3.54 (m, 1H, H-6_b_), 3.38 (d, 1H, *J* = 17.1 Hz, C*H*CN), 2.17, 2.13, 2.08, 2.07, 2.06, 2.01, 1.99 (21H, C*H_3_*COO).

^13^C-NMR (400 MHz, CDCl_3_): δ 171.22, 170.68, 170.53, 170.09, 169.90, 169.72, 169.42 (7C, CH_3_*C*OO), 116.40 (SCH_2_*C*N), 97.36 (C-1′), 83.30 (C-1), 76.85 (C-5), 73.23 (C-3), 69.38 (C-3′), 68.96 (C-5′), 68.86 (C-2′), 68.83 (C-4), 66.70 (C-6), 66.03 (C-4′), 62.41 (C-6′), 52.76 (C-2), 23.14 (CH_3_, NHAc), 20.87, 20.79, 20.72, 20.67, 20.65, 20.61 (6C, *C*H_3_COO), 14.62 (S*C*H_2_CN).

MS: *m*/*z* = 713.10 [M + Na^+^] (calculated 713.18).

Cyanomethyl (2′,3′,4′,6′-Tetra-*O*-acetyl-α-d-mannopyranosyl)-(1→2)-3,4,6-tri-*O*-acetyl-1-thio-α-d-mannopyranoside (**17**)

2,3,4,6-Tetra*-O-*acetyl-α-d-mannopyranosyl trichloracetimidate (**9**) (202 mg, 0.41 mmol, 1 eq.) and cyanomethyl 3,4,6-tri*-O-*acetyl-1-thio-α-d-mannopyranoside (**2b**) (148 mg, 0.41 mmol, 1 eq.) were dissolved in dry dichloromethane (5 mL) in presence of activated molecular sieves and cooled to −63 °C under argon atmosphere. BF_3_OEt_2_ (50.6 μL, 0.41 mmol, 1 eq.) was added, and the mixture was stirred for 30 min. After, the solution was allowed to warm to room temperature and stirred for 1h. The reaction was quenched with triethylamine (57 μL, 0.41 mmol, 1 eq.), stirred for 5 min, filtered over Celite, and concentrated in vacuo. The reaction mixture was monitored by TLC (dichloromethane/acetone 9:1, Rf = 0.42). Column chromatography (SiO_2_, dichloromethane/acetone 9:1) gave the desired product as white solid (149 mg, 52.6%).

^1^H-NMR (400 MHz, CDCl_3_): δ 5.63 (d, 1H, *J* = 1.9 Hz, H-1), 5.45-5.39 (m, 2H, H-4, H-3′), 5.31 (t, 1H, *J* = 9.9 Hz, H-4′), 5.27 (dd, 1H, *J* = 3.4, 1.9, H-2′), 5.16 (dd, 1H, *J* = 9.6, 3.3, H-3), 4.96 (d, 1H, *J* = 1.9 Hz, H-1′), 4.37-4.25 (m, 3H, H-6_a_, H-6_b_, H-5), 4.23-4.12 (m, 4H, H-2, H-5′, H-6′_a_, H-6′_b_), 3.51 (d, 1H, *J* =17.1 Hz, SC*H*_2_CN), 3.40 (d, 1H, *J* = 17.1 Hz, SC*H*_2_CN), 2.17, 2.17, 2.13, 2.12, 2.08, 2.07, 2.04 (7s, 21H, C*H_3_*COO).

^13^C-NMR (400 MHz, CDCl_3_): δ 170.74, 170.65, 170.32, 169.87, 169.74, 169.51, 169.25 (7C, CH_3_*C*OO), 115.75 (SCH_2_*C*N), 99.15 (C-1′), 83.33 (C-1), 76.70 (C-2), 70.35 (C-3), 70.04 (C-5), 69.60, 69.60 (C-2′, C-5′), 68.30, 66.48, 65.95 (C-4, C-4′, C-3′), 62.60 (C-6′), 61.77 (C-6), 20.84, 20.76, 20.68, 20.64, 20.64, 20.62, 20.62 (7C, *C*H_3_COO), 15.91 (S*C*H_2_CN).

MS: *m*/*z* = 714.21 [M + Na^+^] (calculated 714.65).

Propargyl (2′,3′,4′,6′-tetra-*O*-acetyl-α-d-mannopyranosyl)-(1→2)-3,4,6-tri-*O*-acetyl-α-d-mannopyranoside (**18**)

2,3,4,6-Tetra*-O-*acetyl-α-d-mannopyranosyl trichloracetimidate (**9**) (212 mg, 0.43 mmol, 1 eq.) and propargyl 3,4,6-tri*-O-*acetyl-α-d-mannopyranoside (**5b**) (150 mg, 0.43 mmol, 1 eq.) were dissolved in dry dichloromethane (8 mL) in presence of activated molecular sieves. The solution was cooled to −50 °C under argon atmosphere. BF_3_OEt_2_ (49.7 µL, 0.43 mmol, 1 eq.) was added to the flask, and the solution was allowed to warm to room temperature and stirred for 18 h. The reaction was quenched with triethylamine (155 µL, 1.115 mmol, 1 eq.), stirred for 5 min, filtered over Celite, and concentrated in vacuo. The reaction mixture was monitored by TLC (dichloromethane/acetone 9:1, Rf = 0.70). Column chromatography (SiO_2_, dichloromethane/acetone 9:1) gave the desired product as a white solid (232 mg, 80%).

^1^H-NMR (400 MHz, CDCl_3_): δ 5.45-5.27 (m, 5H, H-3′, H-4, H-4′, H-3, H-2′), 5.18 (d, 1H, *J* =2.0 Hz, H-1), 4.95 (d, 1H, *J* = 2.0 Hz, H-1′), 4.29 (d, 2H, *J* = 2.4 Hz, OC*H_2_*C≡CH), 4.28-4.22 (m, 2H, H-6_a_, H-6_b_,), 4.20-4.12 (m, 3H, H-5′, H-6′_a_, H-6′_b_), 4.07(dd, 1H, *J* =1.2, 2.0 Hz H-2), 4.00-3.95 (m, 1H, H-5), 2.51 (t, 1H, *J* = 2.4 Hz, C≡C*H*), 2.17, 2.16, 2.11, 2.10, 2.06, 2.05, 2.03 (7s, 21H, C*H_3_*COO).

^13^C-NMR (101 MHz, CDCl_3_): δ 170.86, 170.54, 170.33, 169.83, 169.70, 169.44, 169.33 (7C, CH_3_*C*OO), 99.20 (C-1′), 96.92 (C-1), 78.11 (OCH_2_*C*≡CH), 76.75 (C-2), 75.54 (OCH_2_C≡*C*H), 70.06 (C-2′), 69.75 (C-3), 69.24 (C-5′), 69.09 (C-5), 68.41 (C-3′), 66.31(C-4′), 66.09 (C-4), 62.35(C-6_a_), 62.03(C-6′), 55.00 (O*C*H_2_C≡CH), 20.90, 20.87, 20.74, 20.71, 20.67, 20.67, 20.65 (7C, *C*H_3_COO).

HRMS: *m*/*z* = 697.1950 [M + Na^+^] (calculated 697.1950).

### 3.8. Enzymatic Deprotection of Disaccharides ***10**–**18***

The deacetylated disaccharides were produced following a general procedure of hydrolysis.

The substrates (10 mM final concentration; 5 mM for **12**) were dissolved in acetonitrile (20%–30% *v*/*v* depending on substrate solubility) under magnetic stirring, and then phosphate buffer (50 mM, pH 4.0–5.8) was added slowly. The reaction was started through the addition of immobilized CRL and/or AXE, previously conditioned with reaction buffer. The reactions were performed at 25 °C under mechanical stirring; the pH of the solution was maintained constant by automatic titration. Reaction course was monitored by TLC.

After complete consumption of the starting substrate or before an excessive formation of undesired products, the reactions were stopped by enzymatic derivative filtration on Büchner funnel. Acetonitrile was evaporated under reduced pressure, and the solution was brined and extracted with ethyl acetate. The organic layers were dried over anhydrous Na_2_SO_4_, filtered, and concentrated in vacuo. The mixture obtained was purified by flash chromatography.

2′,3′,4′,6′-Tetra-*O*-acetyl-α-d-mannopyranosyl-(1→6)-2,3,4-tri-*O*-acetyl-α-d-mannopyranose (**10a**)

2′,3′,4′,6′-Tetra*-O-*acetyl-α-d-mannopyranosyl-(1→6)-1,2,3,4-tetra*-O-*acetyl-α-d-mannopyranose (**10**) was hydrolyzed to the corresponding 2′,3′,4′,6′-tetra*-O-*acetyl-α-d-mannopyranosyl-(1→6)-2,3,4-tri*-O-*acetyl-α-d-mannopyranose (**10a**) following the general procedure for enzymatic hydrolysis: substrate (10 mg, 10 mM) was solubilized in 1.7 mL of phosphate buffer 50 mM pH 4.8 and 30% *v*/*v* of acetonitrile. The reaction was started through addition of AXE-GLX-AG (52 UI). The reaction mixture was monitored by TLC (dichloromethane/methanol 9:1, Rf = 0.76). The product was identified by comparing the reaction mixture with standard material produced in-house [53].

Cyanomethyl (2′,3′,4′,6′-tetra-*O*-acetyl-α-d-mannopyranosyl)-(1→6)-3,4-di-*O*-acetyl-1-thio-α-d-mannopyranoside (**11a**)

Cyanomethyl (2′,3′,4′,6′-tetra*-O-*acetyl-α-d-mannopyranosyl)-2,3,4-tri*-O-*acetyl-1-thio-α-d-mannopyranoside (**11**) was hydrolyzed to the corresponding cyanomethyl (2′,3′,4′,6′-tetra*-O-*acetyl-α-d-mannopyranosyl)-(1→6)-3,4-di*-O-*acetyl-1-thio-α-d-mannopyranoside (**11a**) following the general procedure for enzymatic hydrolysis: substrate (69 mg, 10 mM) was solubilized in 8.5 mL of phosphate buffer 50 mM pH 5.3 and 30% *v*/*v* of acetonitrile. The reaction was started through addition of AXE-GLX-AG (560 UI). The reaction mixture was monitored by TLC (dichloromethane/acetone 8:2, Rf = 0.53). Column chromatography (SiO_2_, dichloromethane/acetone 8:2) gave the desired product as a white sticky solid (15 mg, 23%).

^1^H-NMR (400 MHz, CDCl_3_): δ 5.58 (d, 1H, *J* = 1.4 Hz, H-1), 5.36 (t, 1H, *J* = 9.9 Hz, H-4), 5.33-5.29 (m, 2H, H-4′, H-3′), 5.24 (t, 1H, *J* = 2.2 Hz, H-2′), 5.18 (dd, 1H, *J* = 9.9, 3.2 Hz, H-3), 4.90 (d, 1H, *J* = 1.7 Hz, H-1′), 4.33-4.24 (m, 2H, H-6′_a_, H-5), 4.22 (dd, 1H, *J* = 3.3, 1.4 Hz, H-2), 4.17 (dd, 1H, *J* = 12.2, 2.6 Hz, H-6′_b_), 4.13-4.07 (m, 1H, H-5′), 3.87 (dd, 1H, *J* = 10.8, 7.0 Hz, H-6_a_), 3.60 (dd, 1H, *J* = 10.7, 2.3 Hz, H-6_b_), 3.56 (d, 1H, *J* = 17.3 Hz, SC*H*_2_CN), 3.38 (d, 1H, *J* = 17.3 Hz, SC*H*_2_CN), 2.18, 2.14, 2.12, 2.08, 2.08, 2.02 (5s, 18H, C*H_3_*COO).

^13^C-NMR (400 MHz, CDCl_3_): δ 170.83, 170.22, 170.08, 169.44, 169.81, 169.80 (6C, *C*OO), 115.88 (SCH_2_*C*N), 97.00 (C-1′), 83.33 (C-1), 71.69 (C-3), 70.37 (C-5), 69.53 (C-2, C-2′), 68.92 (C-5′), 68.72, 66.49 (C-4), 65.96 (C-6), 65.88, 62.53 (C-6′), 20.88, 20.80, 20.77, 20.71, 20.67, 20.67 (6C, *C*H_3_COO), 15.21 (S*C*H_2_CN).

MS: *m*/*z* = 672.09 [M + Na^+^] (calculated 672.16).

(4-Methylphenyl) (2′,3′,4′,6′-tetra-*O*-acetyl-α-d-mannopyranosyl)-(1→6)-3,4-di-*O*-acetyl-1-thio-α-d-mannopyranoside (**12a**)

(4-Methylphenyl) (2′,3′,4′,6′-tetra*-O-*acetyl-α-d-mannopyranosyl-(1→6)-2,3,4-tri*-O-*acetyl-1-thio-α-d-mannopyranoside (**12**) was hydrolyzed to the corresponding (4-methylphenyl) (2′,3′,4′,6′-tetra*-O-*acetyl-α-d-mannopyranosyl)-(1→6)-3,4-di*-O-*acetyl-1-thio-α-d-mannopyranoside(**12a**) following the general procedure for enzymatic hydrolysis: substrate (75 mg, 10 mM) was solubilized in 20.37 mL of phosphate buffer 50 mM pH 5.4 and 30% *v*/*v* of acetonitrile. The reaction was started through addition of AXE-GLX-AG (420 UI). The reaction mixture was monitored by TLC (dichloromethane/acetone 9:1, Rf = 0.22). Column chromatography (SiO_2_, dichloromethane/acetone 9:1) gave the desired product as a white sticky solid (17 mg, 24%).

^1^H-NMR (400 MHz, CDCl_3_): δ 7.39 (d, 2H *J* = 8.0 Hz, Ar), 7.17 (d, 2H, *J* = 8.0 Hz, Ar), 5.46 (d, *J* = 1.9 Hz, 1H, H-1), 5.42 (d, 1H, *J* = 9.9 Hz, H-4) 5.36-5.24 (m, 4H, H-2′, H-3′, H-4′, H-3), 4.92 (d, 1H, *J* = 1.6 Hz, H-1′), 4.50 (ddd, 1H, *J* = 10.0, 5.0, 2.4 Hz, H-5), 4.35-4.31 (m, 1H, H-2) 4.28 (dd, 1H, *J* = 12.3, 5.1 Hz, H-6′_a_), 4.09 (dd, 1H, *J* = 12.3, 2.5 Hz, H-6′_b_), 4.03 (ddt, 1H, *J* = 7.5, 5.1, 2.2 Hz, 1H, H-5′), 3.85 (dd, 1H, *J* = 11.4, 5.0 Hz, H-6_a_), 3.62 (dd, 1H, *J* = 11.4, 2.5 Hz, H-6_b_), 2.34 (s, 3H, C*H_3_*-Ar), 2.16, 2.12, 2.11, 2.09, 2.07, 2.02 (6s, 18H, COC*H_3_*).

^13^C-NMR (400 MHz, CDCl_3_): δ 170.72, 170.17, 169.98, 169.76, 169.76, 169.74 (6C, CH_3_*C*OO), 138.07, 132.21, 130.06, 129.39 (Ar), 97.81 (C-1′), 88.06 (C-1), 71.86 (C-3), 70.59 (C-2), 70.28 (C-5), 69.62, 69.00, 68.61 (C-5′), 66.84 (C-6), 66.69 (C-4), 66.05, 62.35 (C-6′), 21.13(CH_3_-Ar), 20.89, 20.89, 20.75, 20.73, 20.73, 20.67 (6C, *C*H_3_COO).

MS: *m*/*z* = 723.24 [M + Na^+^] (calculated 723.19).

Ethyl (2′,3′,4′,6′-tetra-*O*-acetyl-α-d-mannopyranosyl)-(1→6)-3,4-di-*O*-acetyl-1-thio-α-d-mannopyranoside (**13a**)

Ethyl (2′,3′,4′,6′-tetra*-O-*acetyl-α-d-mannopyranosyl)-(1→6)-2,3,4-tri*-O-*acetyl-1-thio-α-d-mannopyranoside (**13**) was hydrolyzed to the corresponding ethyl (2′,3′,4′,6′-tetra*-O-*acetyl-α-d-mannopyranosyl)-(1→6)-3,4-di*-O-*acetyl-1-thio-α-d-mannopyranoside (**13a**) following the general procedure for enzymatic hydrolysis: substrate (28 mg, 10 mM) was solubilized in 4.39 mL of phosphate buffer 50 mM pH 4.8 and 30% *v*/*v* of acetonitrile. The reaction was started through addition of AXE-GLX-AG (160 UI). The reaction mixture was monitored by TLC (dichloromethane/acetone 8:2, Rf = 0.62). Column chromatography (SiO_2_, dichloromethane/acetone 9:1) gave the desired product as a white solid (10 mg, 38%).

^1^H-NMR (400 MHz, CDCl_3_): δ 5.35 (m, 1H, H-1) 5.33-5.22 (m, 5H, H-4, H-4′, H-3′, H-2′,H-3,), 4.90 (d, 1H, *J* = 1.8 Hz, H-1′), 4.40 (ddd, 1H, *J* = 9.3, 6.5, 2.2 Hz, H-5), 4.26-4.14 (m, 3H, H-6′_a_, H-6′_b_, H-2) 4.11 (ddd, 1H, *J* = 9.7, 5.4, 2.4 Hz, 1H, H-5′), 3.83 (dd, 1H, *J* = 10.9, 6.6 Hz, H-6_a_), 3.57 (dd, 1H, *J* = 10.9, 2.2 Hz, H-6_b_), 2.75-2.59 (m, 2H, SC*H*_2_CH_3_), 2.17, 2.13, 2.10, 2.07, 2.06, 2.01 (6s, 18H, COC*H_3_*), 1.35 (t, 3H, *J* = 7.4, SCH_2_CH_3_).

^13^C-NMR (400 MHz, CDCl_3_): δ 170.82, 170.17, 169.94, 169.81, 169.81, 169.81 (6C, CH_3_*C*OO), 97.39 (C-1′), 83.21 (C-1), 72.03, 70.64 (C-2), 69.61, 69.43 (C-5), 68.84, 68.53 (C-5′), 66.96, 66.47 (C-6), 66.15, 62.47 (C-6′), 24.79 (S*C*H_2_CH_3_), 20.90, 20.87, 20.78, 20.74, 20.70, 20.67 (6C, *C*H_3_COO), 14.56 (SCH_2_*C*H_3_).

MS: *m*/*z* = 661.22 [M + Na^+^] (calculated 661.18).

Propargyl (2′,3′,4′,6′-tetra-*O*-acetyl-α-d-mannopyranosyl)-(1→6)-3,4-di-*O*-acetyl-α-d-mannopyranoside (**14a**)

Propargyl (2′,3′,4′,6′-tetra*-O-*acetyl-α-d-mannopyranosyl)-(1→6)-2,3,4-tri*-O-*acetyl-α-d-mannopyranoside (**14**) was hydrolyzed to the corresponding propargyl (2′,3′,4′,6′-tetra*-O-*acetyl-α-d-mannopyranosyl)-(1→6)-3,4-di*-O-*acetyl-α-d-mannopyranoside (**14a**) following the general procedure for enzymatic hydrolysis: substrate (81 mg, 10 mM) was solubilized in 12 mL of phosphate buffer 50 mM pH 4.0 and 30% *v*/*v* of acetonitrile. The reaction was started through addition of AXE-GLX-AG (404 UI). The reaction mixture was monitored by TLC (dichloromethane/acetone 8:2, Rf = 0.41) and purified by flash chromatography (SiO_2_, dichloromethane/acetone 8:2). The desired product was obtained as a as a colorless oil (18.4 mg, 24%).

^1^H-NMR (400 MHz, CDCl_3_): δ 5.38-5.25 (m, 5H, H-4′, H-3′, H-2′, H-4, H-3), 5.10 (d, 1H, *J* = 1.8 Hz, H-1), 4.92 (d, 1H, *J* = 1.8 Hz, H-1′), 4.31 (d, 2H, *J* = 2.4 Hz, OC*H_2_*C≡CH), 4.26 (dd, 1H, *J* = 5.5 Hz, 12.4 Hz, H-6′_a_), 4.19-4.10 (m, 2H, H-6′_b_, H-5′), 4.09 (dd, 1H, *J* = 2.9, 1.9 Hz, H-2), 4.00 (ddd, 1H, *J* = 8.9, 6.0, 2.3 Hz, H-5), 3.81 (dd, 1H, *J* = 5.9 Hz, 11 Hz, H-6_b_), 3.61 (dd, 1H, *J* = 2.4 Hz, 11 Hz, H-6_a_), 2.52 (t, 1H, *J* = 2.4 Hz, C≡C*H*), 2.17, 2.13, 2.11, 2.07, 2.06, 2.01 (6s, 18H, COC*H_3_*).

^13^C-NMR (400 MHz, CDCl_3_): δ 170.78, 170.16, 169.88, 169.88, 169.88, 169.81 (6C, CH_3_*C*OO), 97.86 (C-1), 96.35 (C-1′), 78.32 (OCH_2_*C*≡CH), 75.36 (OCH_2_C≡*C*H), 71.48, 69.83 (H-5), 69.58, 69.18, 68.99 (C-2), 68.65 (C-5′), 66.67 (C-6), 66.60, 66.04, 62.46 (C-6′), 54.82 (O*C*H_2_C≡CH), 20.88, 20.88, 20.77, 20.72, 20.72, 20.68 (6C, *C*H_3_COO).

MS: *m*/*z* = 655.58 [M + Na^+^] (calculated 655.19).

Allyl (2′,3′,4′,6′-tetra-*O*-acetyl-α-d-mannopyranosyl)-(1→6)-3,4-di-*O*-acetyl-α-d-mannopyranoside (**15a**)

Allyl (2′,3′,4′,6′-tetra*-O-*acetyl-α-d-mannopyranosyl)-(1→6)-2,3,4-tri*-O-*acetyl-α-d-mannopyranoside (**15**) was hydrolyzed to the corresponding Allyl (2′,3′,4′,6′-tetra*-O-*acetyl-α-d-mannopyranosyl)-(1→6)-3,4-di*-O-*acetyl-α-d-mannopyranoside (**15a**) following the general procedure for enzymatic hydrolysis: substrate (103 mg, 10 mM) was solubilized in 15.2 mL of phosphate buffer 50 mM pH 4.8 and 30% *v*/*v* of acetonitrile. The reaction was started through addition of AXE-GLX-AG (504 UI). The reaction mixture was monitored by TLC (dichloromethane/acetone 8:2, Rf = 0.33). Column chromatography (SiO_2_, dichloromethane/acetone 8:2,) gave the desired product as a white solid (50 mg, 52%).

^1^H-NMR (400 MHz, 45 °C, CDCl_3_): δ 5.94 (dddd, 1H, *J* = 17.2, 10.4, 6.2, 5.3, CH_2_C*H*=CH_2_), 5.39-5.24 (m, 7H, H-3, H-4, H-2′, H-3′, H-4′, CH_2_CH=C*H_2_*), 4.91 (d, 1H, *J* = 1.8 Hz, H-1) 4.90 (d, 1H, *J* = 1.8 Hz, H-1′), 4.28-4.21 (m, 2H, H-6′_a_, C*H_2_*CH=CH_2_), 4.16 (dd, 1H, *J* = 7.9, 2.3 Hz, H-6′_b_), 4.15-4.03 (m, 3H, H-2, H-5′, C*H_2_*CH=CH_2_), 3.99 (ddd, 1H, *J* = 9.9, 6.1, 2.4 Hz, H-5), 3.80 (dd, 1H, *J* = 10.9, 6.2 Hz, H-6_a_), 3.58 (dd, 1H, *J* = 10.9, 2.3 Hz, H-6_b_), 2.17, 2.12, 2.10, 2.05, 2.06, 2.00 (6s, 18H, C*H*_3_COO).

^13^C-NMR (400 MHz, CDCl_3_): δ 170.79, 170.15, 169.94, 169.93, 169.83, 169.80 (6C, CH_3_*C*OO), 133.25 (CH_2_*C*H=CH_2_), 118.23 (CH_2_CH=*C*H_2_), 98.19 (C-1), 97.25 (C-1′), 71.73, 69.62, 69.35, 69.29, 68.91, 68.60, 68.40, 66.76, 66.63, 66.11, 62.46 (11C, carbon ring), 20.90, 20.89, 20.77, 20.74. 20.70, 20.67 (6C, *C*H_3_COO).

MS: *m*/*z* = 657.25 [M + Na^+^] (calculated 657.20).

Cyanomethyl (3′,4′,6′-tri-*O*-acetyl-α-d-mannopyranosyl)-(1→6)-2-acetamido-3,4-di-*O*-acetyl-2-deoxy-1-thio-β-d-glucopyranoside (**16a**)

Cyanomethyl (2′,3′,4′,6′-tetra*-O-*acetyl-α-d-mannopyranosyl)-(1→6)-2-acetamido-3,4-di*-O-*acetyl-2-deoxy-1-thio-β-d-glucopyranoside (**16**) was hydrolyzed to the corresponding cyanomethyl (3′,4′,6′-tri*-O-*acetyl-α-d-mannopyranosyl)-(1→6)-2-acetamido-3,4-di*-O-*acetyl-2-deoxy-1-thio-β-d-glucopyranoside (**16a**) following the general procedure for enzymatic hydrolysis: substrate (80.5 mg, 10 mM) was solubilized in 11.6 mL of phosphate buffer 50 mM pH 4.5 and 30% *v*/*v* of acetonitrile. The reaction was started through addition of AXE-GLX-AG (378 UI). The reaction mixture was monitored by TLC (dichloromethane/methanol 9:1, Rf = 0.42). Column chromatography (SiO_2_, dichloromethane/acetone 8:2) gave the desired product as a white sticky solid (15.4 mg 20.5%).

^1^H-NMR (400 MHz, CDCl_3_): δ 6.02 (d, 1H, *J* = 9.3 Hz, NH), 5.39–5.07 (m, 4H, H-4′, H-3′, H-3, H-4), 4.95 (d, 1H, *J* = 1.9 Hz, H-1′), 4.75 (d, 1H, *J* = 10.4 Hz, H-1), 4.29 (dd, 1H, *J* = 12.3, 4.9 Hz, H-6′_a_), 4.25-4.10 (m, 3H, H-2, H-2′, H-6′_b_), 3.98 (ddd, 1H, *J* = 9.9, 4.9, 2.4 Hz, H-5′) 3.85-3.64 (m, 4H, C*H*CN, H-6_a_, H-5, H-6_b_), 3.34 (d, 1H, *J* = 17.3 Hz, C*H*CN), 2.12, 2.10, 2.08, 2.07, 2.05, 1.99 (6s, 18H, C*H_3_*COO).

^13^C-NMR (400 MHz, CDCl_3_): δ 171.33, 170.84, 170.60, 170.01, 169.84, 169.26 (6C, CH_3_COO), 116.53 (SCH_2_*C*N), 99.63 (C-1′), 83.36 (C-1), 77. 18 (C-5), 73.46 (C-3), 71.35 (C-3′), 68.92 (C-5′), 68.78 (C-2′), 68.67 (C-4), 66.23 (C-6), 65.95 (C-4′), 62.48 (C-6′), 52.57 (C-2), 23.11 (CH_3_, NHAc), 20.90, 20.81, 20.4, 20.66, 20.64 (5C, CH_3_COO), 14.50 (S*C*H_2_CN).

MS: *m*/*z* = 671.21 [M + Na^+^] (calculated 671.17).

Cyanomethyl (2′,3′,4′,6′-tetra-*O*-acetyl-α-d-mannopyranosyl)-(1→2)-3,4-di-*O*-acetyl-1-thio-α-d-mannopyranoside (**17a**)

Cyanomethyl (2′,3′,4′,6′-tetra*-O-*acetyl-α-d-mannopyranosyl)-(1→2)-3,4,6-tri*-O-*acetyl-1-thio-α-d-mannopyranoside (**17**) was hydrolyzed to the corresponding cyanomethyl (2′,3′,4′,6′-tetra*-O-*acetyl-α-d-mannopyranosyl)-(1→2)-3,4-di*-O-*acetyl-1-thio-α-d-mannopyranoside (**17a**) following the general procedure for enzymatic hydrolysis: substrate (95 mg, 10 mM) was solubilized in 13.10 mL of phosphate buffer 50 mM pH 5.0 and 30% *v*/*v* of acetonitrile. The reaction was started through addition of AXE-GLX-AG (477 UI). The reaction mixture was monitored by TLC (dichloromethane/acetone 8:2, Rf = 0.33). Column chromatography (SiO_2_, dichloromethane/acetone 8:2) gave the desired product as a white solid (14.5 mg, 16%).

^1^H-NMR (400 MHz, CDCl_3_): δ 5.55 (d, 1H, *J* = 1.8 Hz, H-1), 5.30 (dd, 1H, *J* = 9.9, 3.4 Hz, H-3′), 5.23 (t, 2H, *J* = 10.3 Hz, H-4, H-4′), 5.18 (dd, 1H, *J* = 3.4, 1.9, H-2′), 5.11 (dd, 1H, *J* = 9.7, 3.3, H-3), 4.86 (d, 1H, *J* = 1.9 Hz, H-1′), 4.22 (dd, 1H, H-6′_a_), 4.16-4.09 (m, 2H, H-2, H-5′), 4.06 (dd, 1H, *J* = 11.9, 2.9 Hz, H-6′_b_), 4.00 (m, broad, 1H, *J* = 10.1, 4.5, 2.4 Hz, H-5), 3.75-3.58 (m, 2H, H-6_a_, H-6_b_), 3.41 (d, 1H, *J* = 17.2 Hz, SC*H*_2_CN), 3.31 (d, 1H, *J* = 17.2 Hz, SC*H*_2_CN), 2.08, 2.05, 2.04, 2.02, 2.01, 1.95 (6s, 18H, C*H_3_*COO).

^13^C-NMR (400 MHz, CDCl_3_): δ 170.88, 170.36, 170.34, 169.91, 169.91, 169.67 (6C, CH_3_*C*OO), 116.06 (SCH_2_*C*N), 99.06 (C-1′), 83.49 (C-1), 76.86 (C-2), 72.27 (C-5), 70.27 (C-3), 69.51, 69.41 (C-5′, C-2′), 68.46 (C-3′), 66.46, 65.87 (C-4, C-4′), 62.83 (C-6′), 61.07 (C-6), 20.86, 20.84, 20.74, 20.71, 20.64, 20.61 (6C, *C*H_3_COO), 15.02 (S*C*H_2_CN).

MS: *m*/*z* = 672.09 [M + Na^+^] (calculated 672.16).

Propargyl (2′,3′,4′,6′-tetra-*O*-acetyl-α-d-mannopyranosyl)-(1→2)-3,4-di-*O*-acetyl-α-d-mannopyranoside (**18a**)

Propargyl (2′,3′,4′,6′-tetra*-O-*acetyl-α-d-mannopyranosyl)-(1→2)-3,4,6-tri*-O-*acetyl-α-d-mannopyranoside (**18**) was hydrolyzed to the corresponding propargyl (2′,3′,4′,6′-tetra*-O-*acetyl-α-d-mannopyranosyl)-(1→2)-3,4-di*-O-*acetyl-α-d-mannopyranoside (**18a**) following the general procedure for enzymatic hydrolysis: substrate (38 mg, 10 mM) was solubilized in 5.6 mL of phosphate buffer 50 mM pH 5.8 and 30% *v*/*v* of acetonitrile. The reaction was started through addition of AXE-GLX-AG (102 UI). The reaction mixture was monitored by TLC (dichloromethane/acetone 8:2). Column chromatography (SiO_2_, dichloromethane/acetone 9:1, Rf = 0.17) gave the desired product as a white solid (7 mg, 20%).

^1^H-NMR (400 MHz, CDCl_3_): δ 5.40 (dd, 1H, *J* = 10.0, 3.3 Hz, H-3′), 5.37-5.26 (m, 4H, H-3, H-4, H-4′, H-2′), 5.19 (d, 1H, *J* = 2.0 Hz, H-1), 4.95 (d, 1H, *J* = 1.9 Hz, H-1′), 4.29 (d, 2H, *J* = 2.4 Hz, OC*H_2_*C≡CH), 4.27-4.15 (m, 3H, H-5′, H-6′_a_, H-6′_b_,), 4.10 (dd, 1H, *J* = 3.2, 2.0 Hz, H-2), 3.78 (ddd, 1H, *J* = 9.6, 4.4, 2.3 Hz, H-5), 3-75-3.71 (m, 2H, H-6_a_, H-6_b_), 2.50 (t, 1H, *J* = 2.4 Hz, OCH_2_C≡C*H*), 2.17, 2.13, 2.11, 2.09, 2.08, 2.03 (6s, 18H, C*H_3_*COO).

^13^C-NMR (400 MHz, CDCl_3_): δ 170.91, 170.39, 170.35, 169.92, 169.89, 169.63 (6C, CH_3_*C*OO), 99.09 (C-1′), 96.86 (C-1), 78.20 (OCH_2_*C*≡CH), 76.75 (C-2), 75.53 (OCH_2_C≡*C*H), 71.33 (C-5), 69.96, 69.64, 69.13 (C-5′), 68.60 (C-3′), 66.58, 66.29, 62.65 (C-6′), 61.28 (C-6), 54.89 (O*C*H_2_C≡CH), 20.87, 20.83, 20.73, 20.73, 20.73, 20.67, (6C, *C*H_3_COO).

MS: *m*/*z* = 655.20 [M + Na^+^] (calculated 655.19).

## 4. Conclusions

By using immobilized CRL or AXE, a library of acetylated mannose-based monosaccharide building blocks, selectively deprotected in position C6 or C2, was prepared. The proposed biocatalytic approach is versatile and simple since the building blocks can be obtained directly by enzymatic hydrolysis, starting from substrates with different reactive group/linker in anomeric position and using acetyl as the only protecting group in the other positions. Interestingly, AXE was also able to provide different acetylated disaccharides, selectively deprotected in position C2 or C6, when C2 position was involved in the glycosidic bond. All the products prepared are potentially useful in the synthesis of various oligosaccharides and their glycoconjugate derivatives.

## Figures and Tables

**Table 1:** Immobilization screening.

Immobilization Carrier	Temperature (°C)	Time (h)	Loading ^b^ (mg/g)	Immobilized Protein (%)	Immobilized Activity (%)	Activity (U/g)
Acrylic resin ^a^	25	24	150	39	38	107
GLX-AG	4	18	150	26	48	283
GLX-AG	25	3	150	28	33	383
GLX-AG	25	3	50	61	43	392

^a^ Sepabeads EC-EP/M from Resindion. ^b^ Quantity of protein used in the immobilization process per gram of carrier.

**Table 2:** Regioselective enzymatic hydrolysis of monosaccharides.

Substrate	Enzyme	Time (h)	Position	Product (Yield)		By-Products
**1**	CRL	21	6OH 1OH	**1a** **1b**	(77%) (13%)	(10%)
	AXE	24	1OH	**1b**	(50%)	-
**2**	CRL	20	6OH 2OH	**2a** **2b**	(50%) (40%)	(10%)
	AXE	72	6OH	**2a**	(26%)	(74%)
**3**	CRL	48	6OH	**3a**	(65%)	(35%)
	AXE	72h			(27%)	(73%)
**4**	CRL	24h	6OH	**4a**	(69%)	(31%)
	AXE	48h			(24%)	(76%)
**5**	CRL	20h	6OH 2OH	**5a** **5b**	(80%) (8%)	(12%)
	AXE	24h	6OH 2OH	**5a** **5b**	(5%) (40%)	(55%)
**6**	CRL	6h	6OH	**6a**	(81%)	(19%)
	AXE	6h			(24%)	(48%)
**7**	CRL	24h	6OH	**7a**	(70%)	(30%)
	AXE	24h	-	-	-	-
**8**	CRL AXE	48h 24h	6OH	**8a**	(68%) (90%)	(32%) (10%)

Experimental conditions: 50 mM KH_2_PO_4_ pH 4.0 (except for compound **8** pH = 5.0), 20%–30% *v*/*v* acetonitrile, room temperature, substrate concentration = 10 mM, CRL immobilized on OC-AG and AXE immobilized on GLX-AG.

**Table 3:** Reaction conditions for disaccharides synthesis.

Compound	Temperature (°C)	Reaction Time (h)	Yield (%)
**10**	0	2.5	87
**11**	−70 to r.t.	4	79
**12**	0	3.5	92
**13**	0	4	37
**14**	0	4	90
**15**	0	2	50
**16**	0	2.5	74
**17**	−63 to r.t.	1.5	52
**18**	−50 to r.t.	18	80

**Table 4:** Regioselective enzymatic deprotection of mannose-based disaccharides (**10**–**18**) using AXE-GLX-AG.

Substrate	Time (h)	Position	Product	Yield (%) ^a^	Conversion (%) ^b^
**10**	48	1OH	**10a**	50	63
**11**	5	2OH	**11a**	23	53
**12**	57	2OH	**12a**	24	60
**13**	7	2OH	**13a**	38	41
**14**	26	2OH	**14a**	24	85
**15**	15	2OH	**15a**	52	84
**16**	168	2′OH	**16a**	21	40
**17**	8.5	6OH	**17a**	16	46
**18**	24	6OH	**18a**	20	32

Experimental conditions: 25 mM KH_2_PO_4_ pH 4.0–5.8, 30%–35% *v*/*v* acetonitrile, room temperature, substrate concentration = 5–10 mM, AXE immobilized on GLX-AG. ^a^ Yield was determined by isolation and purification by flash chromatography. ^b^ Conversion was calculated based on the isolated amount of unconverted substrate.
